# Supplementary material for: Bacteria employ lysine acetylation of transcriptional regulators to adapt gene expression to cellular metabolism
Source: Nat Commun. 2024 Feb 23;15:1674. doi: 10.1038/s41467-024-46039-8 (PMC10891134; doi:10.1038/s41467-024-46039-8)
Supplement: Supplementary file 1 — Supplementary Information [file 41467_2024_46039_MOESM1_ESM.pdf]

## SUPPLEMENTARY INFORMATION

# Bacteria employ lysine acetylation of transcriptional regulators to adapt gene expression to cellular metabolism

Magdalena Kremer[1,2,6], Sabrina Schulze[2,6], Nadja Eisenbruch[2], Felix Nagel[3], Robert Vogt[2], Leona Berndt[2], Babett Dörre[2], Gottfried J. Palm[2], Jens Hoppen[2], Britta Girbardt[2], Dirk Albrecht[4], Susanne Sievers[4], Mihaela Delcea[3,] Ulrich Baumann[1], Karin Schnetz[5] and Michael Lammers[2*]

[1]Institute of Biochemistry, University of Cologne, Zülpicher Straße 47, 50674 Cologne, Germany
[2]Institute of Biochemistry, Department of Synthetic and Structural Biochemistry, University of Greifswald, Felix-Hausdorff-Str. 4, 17489 Greifswald, Germany
[3]Institute of Biochemistry, Department of Biophysical Chemistry, University of Greifswald, Felix-Hausdorff-Str. 4, 17489 Greifswald, Germany
[4]Institute of Microbiology, Department of Microbial Physiology and Molecular Biology, University of Greifswald, Felix-Hausdorff-Str. 8, 17489 Greifswald, Germany
[5]Institute for Genetics, University of Cologne Zülpicher Straße 47a, 50674 Cologne, Germany

[6]: These authors contributed equally to this article.
[*]Correspondence should be addressed to Michael Lammers; Email: michael.lammers@uni-greifswald.de; Tel.: 03834-420-4356; Fax: 03834-420-4373; ORCID: 0000-0003-4168-4640

**Keywords:** RutR, transcriptional regulator, pyrimidine, lysine acetylation, genetic code expansion concept

**Running title:** RutR is regulated by post-translational lysine acetylation

Supplementary Figure 1-15

Supplementary Table 1-12

a

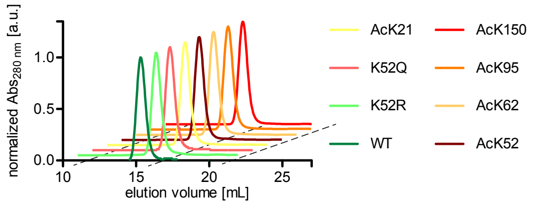


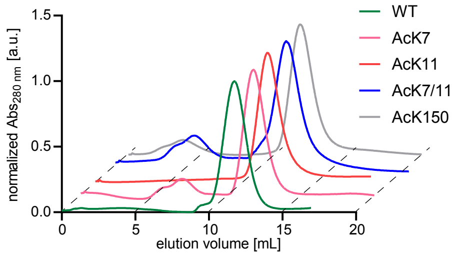


| RutR-His6 | expected molecular weight [Da] | elution volume [mL] | calculated molecular weight [Da] | oligomeric state |
|---|---|---|---|---|
| WT | 24568 | 15.32 | 54920 | 2.23 |
| K52R | 24596 | 15.37 | 53655 | 2.18 |
| K52Q | 24567 | 15.31 | 55176 | 2.25 |
| AcK21 | 24610 | 15.36 | 53906 | 2.19 |
| AcK52 | 24610 | 15.32 | 54920 | 2.23 |
| AcK62 | 24610 | 15.36 | 53906 | 2.19 |
| AcK95 | 24610 | 15.29 | 55693 | 2.27 |
| AcK150 | 24610 | 15.30 | 55434 | 2.26 |

| RutR His6 | expected molecular weight [Da] | elution volume [mL] | calculated molecular weight [Da] | oligomeric state |
|---|---|---|---|---|
| WT | 24568 | 11.67 | 59950 | 2.44 |
| AcK7 | 24610 | 11.68 | 59616 | 2.42 |
| AcK11 | 24610 | 11.64 | 60367 | 2.45 |
| AcK7/11 | 24652 | 11.58 | 61510 | 2.50 |
| AcK150 | 24610 | 11.63 | 60556 | 2.46 |

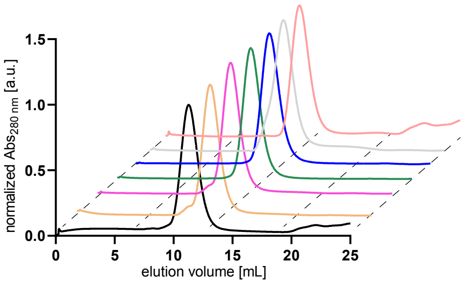


| RutR His6 | expected molecular weight [Da] | elution volume [mL] | calculated molecular weight [Da] | oligomeric state |
|---|---|---|---|---|
| WT | 24568 | 11.29 | 55887 | 2.27 |
| K7Q | 24567 | 11.30 | 55697 | 2.27 |
| K11Q | 24567 | 11.30 | 55887 | 2.27 |
| K7R | 24596 | 11.36 | 54569 | 2.22 |
| K11R | 24596 | 11.36 | 54569 | 2.22 |
| K7Q/K11Q | 24567 | 11.31 | 55507 | 2.26 |
| K7R/K11R | 24624 | 11.38 | 54198 | 2.20 |

b

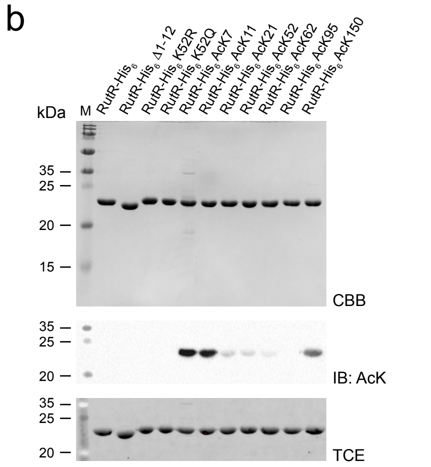


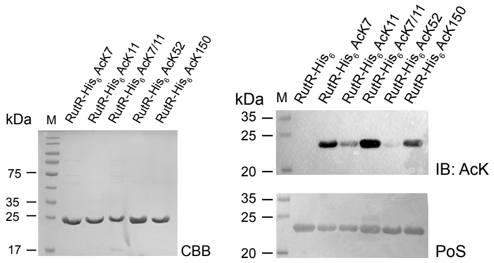


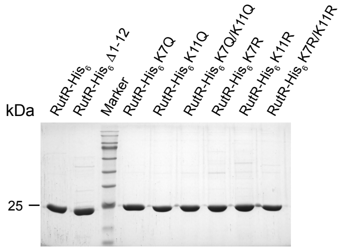


**Supplementary Figure 1: Purification, immunoblotting and analytical size-exclusion chromatography (SEC) of RutR variants RutR AcK7, AcK11, AcK7/11, AcK21, AcK52, AcK62, AcK95 and AcK150 and RutR Δ1-12.**

**a.** Analytical size-exclusion chromatography (SEC) of all full-length RutR variants. Upper panels: Overlay of SEC chromatograms retrieved from protein analysis on a Superdex 200 Increase 10/300 GL column performed separately with 300 µg of each RutR variant. Lower panels: Elution volumes and calculated molecular weights in comparison to expected molecular weights are used to calculate an oligomeric state. The calculated molecular weights were obtained based on the elution volume using a calibration curve.

**b.** SDS-PAGE and immunoblot of all purified RutR variants. 2-5 µg of each purified RutR variant was separated in an SDS-PAGE (12 bzw. 16% acrylamide) and stained using Coomassie Brilliant Blue (CBB). 2-5 µg of each purified RutR variant was separated on a SDS-PAGE (12 bzw. 16% acrylamide) and subsequently subjected to immunoblotting (IB). Probing with anti-AcK-antibody was followed by stripping of the membrane and probing with anti-His6-AB while total protein staining with 2,2,2-trichloroethanol (TCE) or Ponceau S red staining of the membrane was used as loading control. Source data are provided as Source Data file.

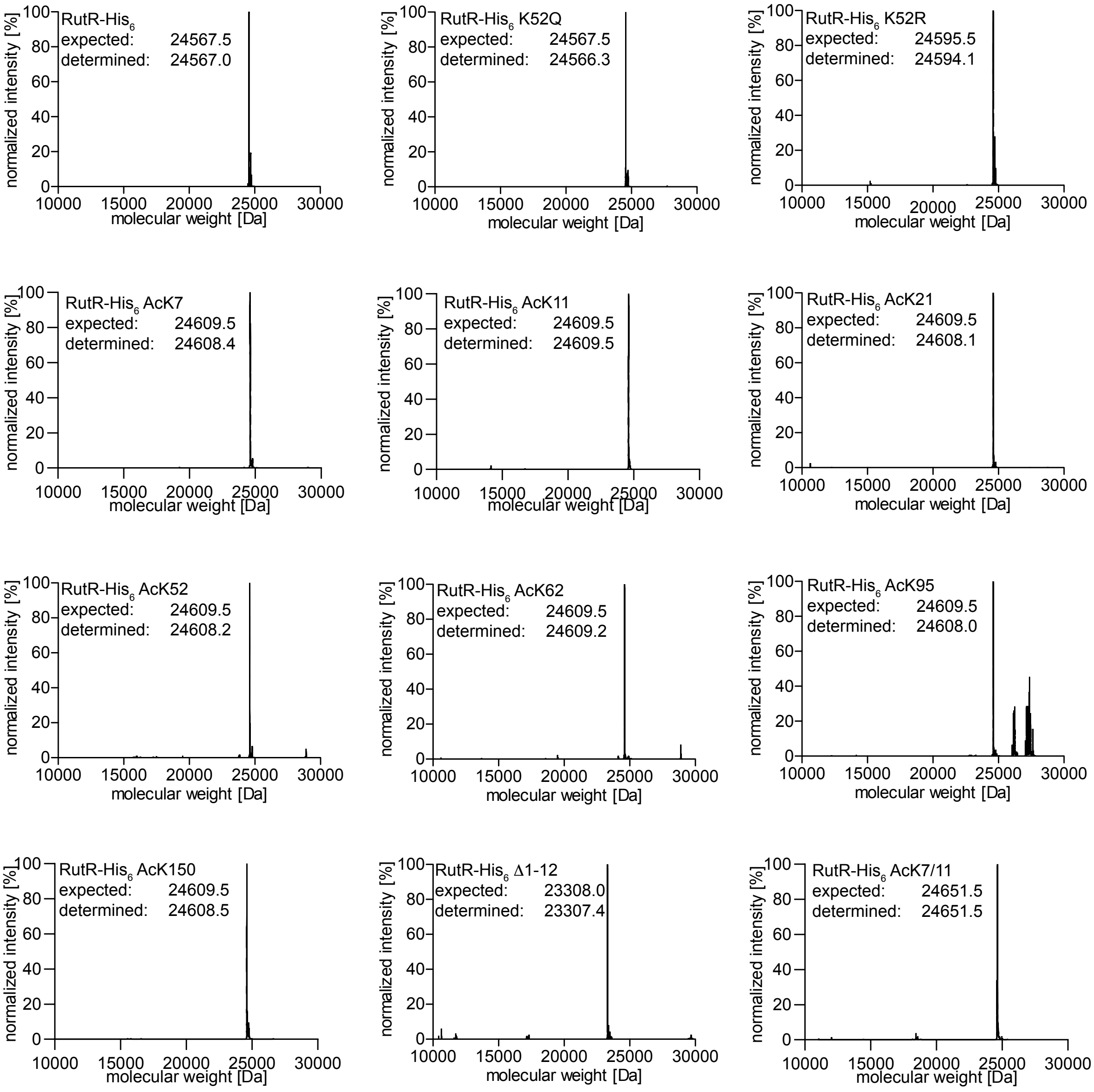


| RutR-His$_6$ | WT | Δ1-12 | K52Q | K52R | AcK7 | AcK11 | AcK21 | AcK52 | AcK62 | AcK95 | AcK150 | AcK7/11 |
|---|---|---|---|---|---|---|---|---|---|---|---|---|
| expected molecular weight [Da] | 24567.5 | 23308.0 | 24567.5 | 24595.5 | 24609.5 | 24609.5 | 24609.5 | 24609.5 | 24609.5 | 24609.5 | 24609.5 | 24651.5 |
| determined molecular weight [Da] | 24567.0 | 23307.4 | 24566.3 | 24594.1 | 24608.4 | 24609.5 | 24608.1 | 24608.2 | 24609.2 | 24608.0 | 24608.5 | 24651.5 |

**Supplementary Fig 2: Quantitative incorporation of N-(ε)-acetyl-L-lysine into RutR at distinct sites shown by electrospray-ionization mass-spectrometry (ESI-MS).**
Electrospray-ionization mass-spectrometry (ESI-MS) of RutR proteins. The determined molecular masses do correspond exactly (≤ 1.5 Da) to the protein masses (RutR: 24567.5 Da; acetylated RutR: 24609.5 Da; acetyl group: 42 Da). All RutR proteins, i.e. lysine-acetylated and non-acetylated RutR, carry a C-terminal hexahistidine-tag (His$_6$-tag). The table summarizes the expected and determined molecular weights of the proteins.

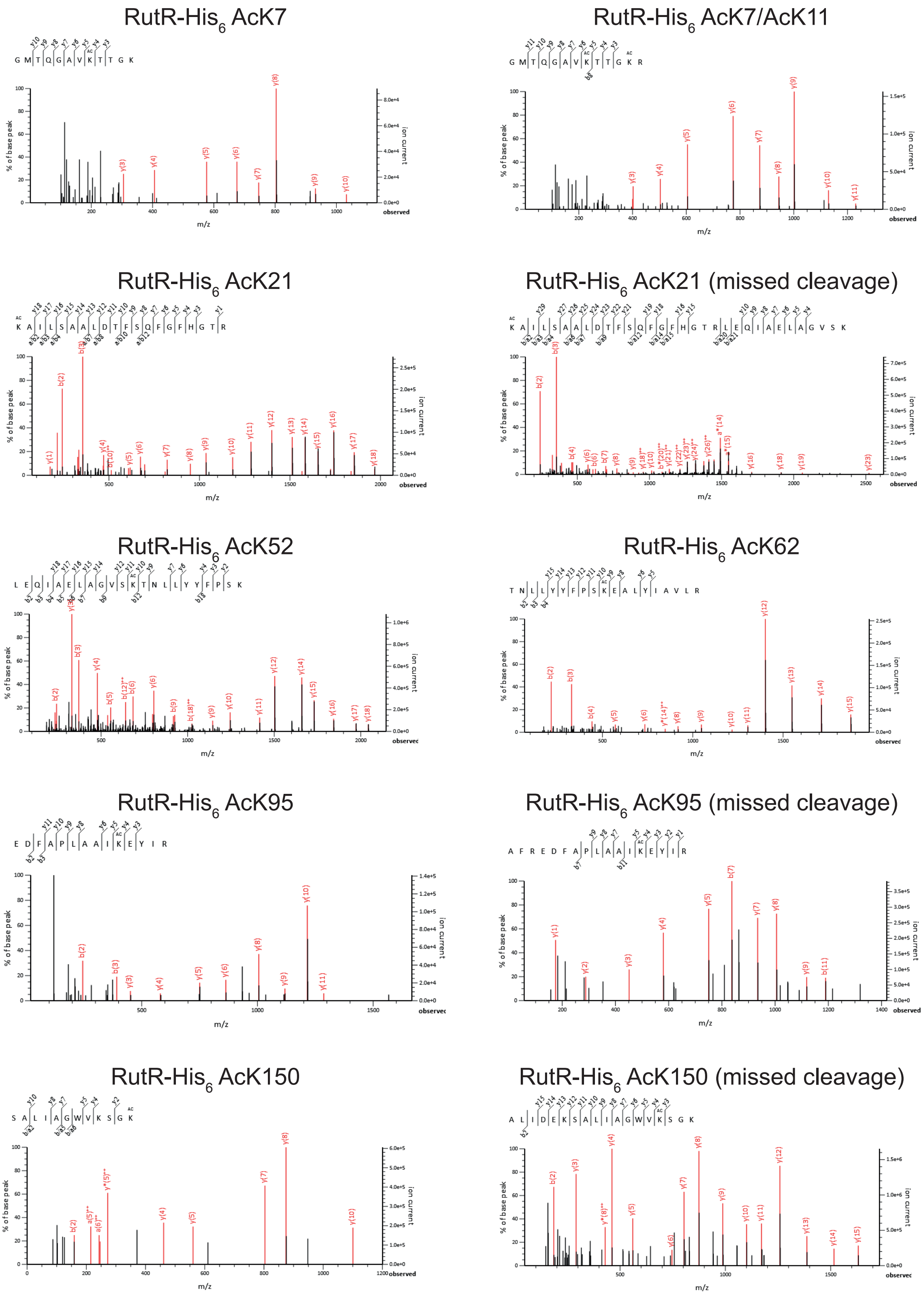


**Supplementary Figure 3: LC-MS/MS fragmentation spectra of lysine-acetylated peptides obtained for recombinantly expressed and purified site-specifically lysine-acetylated RutR.** Except for RutR AcK11 all acetylation sites were confirmed. For RutR Ack21, Ack95 and AcK150 two peptides were identified one of which containing a missed cleavage. RutR AcK11 was not detectable the peptide resulting after proteolytic cleavage was too small (8-TTGKR-12). However, as the total mass of RutR AcK11 was correct, sequencing of the DNA-construct revealed presence of an amber stop codon at the correct position and the immunoblotting by anti-AcK antibody showed that it is lysine-acetylated, the site-specific acetylation of RutR at K11 was confirmed.

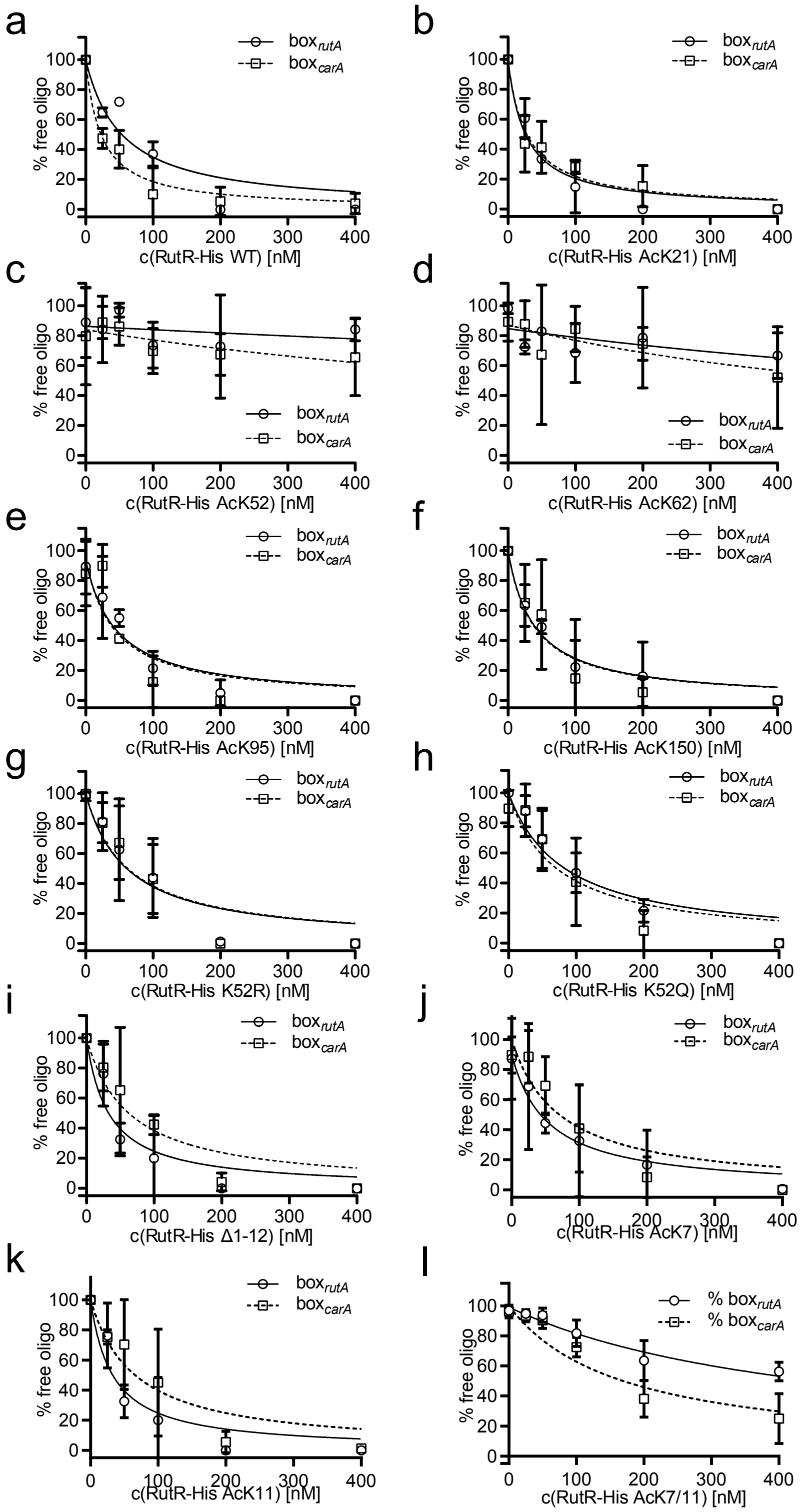


| RutR-$His_6$ | $K_D$ ($box_{rutA}$) [nM] | $K_D$ ($box_{carA}$) [nM] |
|---|---|---|
| non-acetylated | 54 | 23 |
| AcK7 | 53 | 74 |
| AcK11 | 74 | 66 |
| AcK21 | 26 | 29 |
| AcK52 | 3706 | 1131 |
| AcK62 | 1335 | 741 |
| AcK95 | 46 | 43 |
| AcK150 | 39 | 38 |
| K52R | 61 | 63 |
| K52Q | 83 | 71 |
| Δ1-12 | 63 | 54 |
| AcK7/11 | 462 | 169 |

**Supplementary Figure 4: Quantification of the electrophoretic-mobility shift assays (EMSAs).**
**a-l.** Protein-DNA interaction between RutR variants and promoter ($box_{rutA}$: 50 bp segment of $Prom_{rutA}$, $box_{carA}$:46 bp dsDNA segment of $Prom_{carA}$) as well as control (ctrl) oligonucleotides were studied in EMSAs. The signal intensities were quantified using ImageJ and plotted as a function of the RutR protein concentration. The values are given as means ± standard deviations calculated from at least three replicates ($n$≥3). The binding curves were analysed by applying a quadratic model to fit the data. The table summarizes the $K_D$ values as indicated. Source data are provided as Source Data file.

a

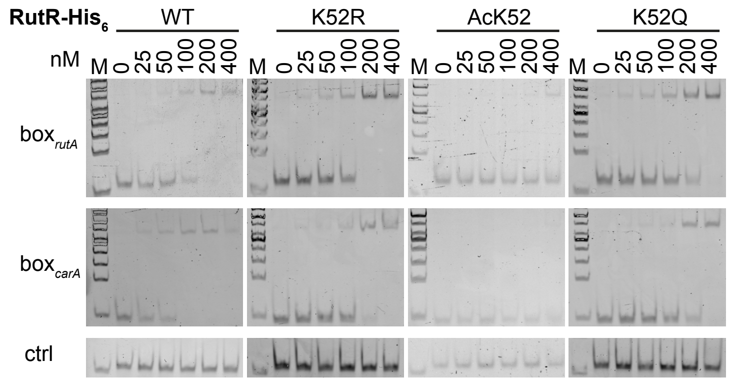


b

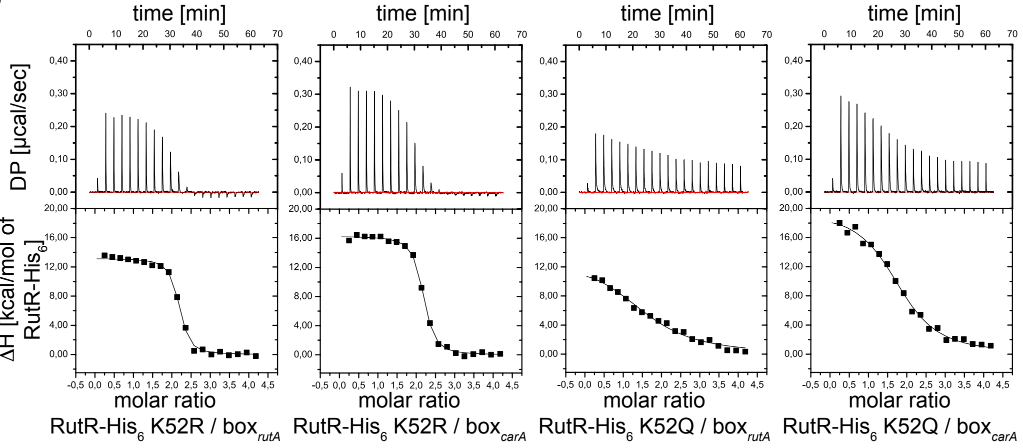


c

| | box$_{rutA}$ | | | | | box$_{carA}$ | | | | |
|---|---|---|---|---|---|---|---|---|---|---|
| RutR-His$_6$ | WT | K52R | K52Q | AcK52 | AcK62 | WT | K52R | K52Q | AcK52 | AcK62 |
| $K_D$ [nM] | 43.7 ± 20.6 | 42.3 ± 30.2 | 2424.5 ± 961.3 | no binding | no binding | 28.0 ± 1.0 | 60.6 ± 35.5 | 1218.6 ± 1129.8 | no binding | 1123.8 ± 108.2 |
| N | 2.24 ± 0.10 | 2.01 ± 0.19 | 1.84 ± 0.14 | no binding | no binding | 2.13 ± 0.07 | 1.93 ± 0.18 | 1.95 ± 0.19 | no binding | 1.58 ± 0.18 |
| ΔH [kcal/mol] | 10.50 ± 1.07 | 13.00 ± 1.47 | 15.83 ± 5.78 | no binding | no binding | 15.36 ± 0.52 | 15.35 ± 0.79 | 17.28 ± 7.50 | no binding | 14.26 ± 1.57 |
| TΔS [kcal/mol] | 20.41 ± 0.89 | 23.01 ± 1.16 | 23.33 ± 5.47 | no binding | no binding | 25.48 ± 0.51 | 25.11 ± 0.76 | 25.39 ± 6.96 | no binding | 22.24 ± 1.57 |

**Supplementary Figure 5: Glutamine is a poor molecular mimic for RutR K52-acetylation as shown by EMSAs and by isothermal titration calorimetry (ITC).**

**a.** Protein-DNA interaction between RutR variants and promoter (box$_{rutA}$: 50 bp segment of Prom$_{rutA}$, box$_{carA}$: 46 bp dsDNA segment of Prom$_{carA}$) as well as control (ctrl) oligonucleotides were studied in EMSAs. Mutation of K to R is often used to conserve a non-acetylated, positively-charged state and mutation of K to Q to mimic a lysine acetylation. However, apart from the neutral charge a Q does sterically not resemble an AcK. Sterically a mutation of K to R might mimic an AcK more than a Q. One exemplary result of at least three replicates is shown (*n*=3). Source data are provided as Source Data file.

**b.** Interaction between RutR K52R and K52Q mutants towards box$_{rutA}$ and box$_{carA}$ DNA analyzed by ITC. Shown are exemplary ITC traces (DP: differential power). A one-site binding model was used to fit the data. All interactions were determined at least in three biologically independent experiments and the values are given as means ± standard deviations (Supplementary Table 3; Source Data file) (*n*≥3).

**c.** Summary of the thermodynamic characterization of the interaction of acetylated RutR AcK52 and AcK62 and RutR K52R/K52Q mutants towards box$_{rutA}$ and box$_{carA}$ DNA analyzed by ITC. Shown are the results of all measurements. A one-site binding model was used to fit the data. All interactions were determined at least in three biologically independent experiments and the values are given as means ± standard deviations (Supplementary Table 3; Source Data file) (*n*≥3). Source data are provided as Source Data file.

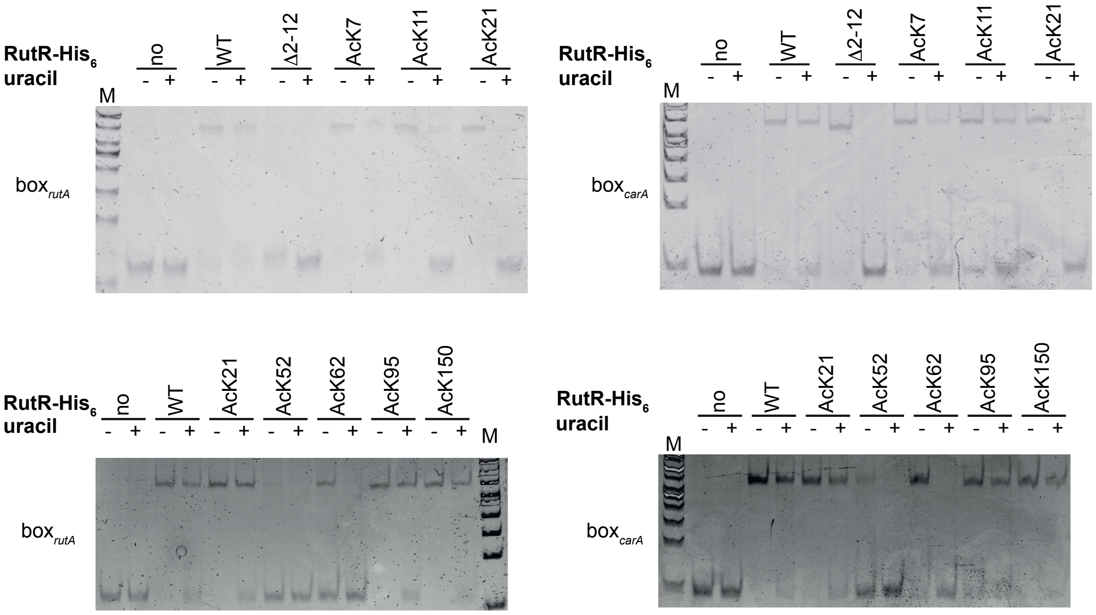


**Supplementary Figure 6: Uracil and RutR acetylation are additively impairing DNA-binding.** Electrophoretic-mobility shift assays were performed to assess the impact of uracil on binding of acetylated RutR variants to box$_{carA}$ and box$_{rutA}$ DNA. Presence of 200 $\mu$M uracil (+) impairs RutR DNA-binding to both DNA fragments. For RutR AcK7, AcK11, AcK52 and AcK62 addition of uracil results in an additional decrease of RutR DNA-binding suggesting that acetylation and uracil exert their impact using different molecular mechanisms. Experiment was conducted once (*n*=1). Source data are provided as Source Data file.

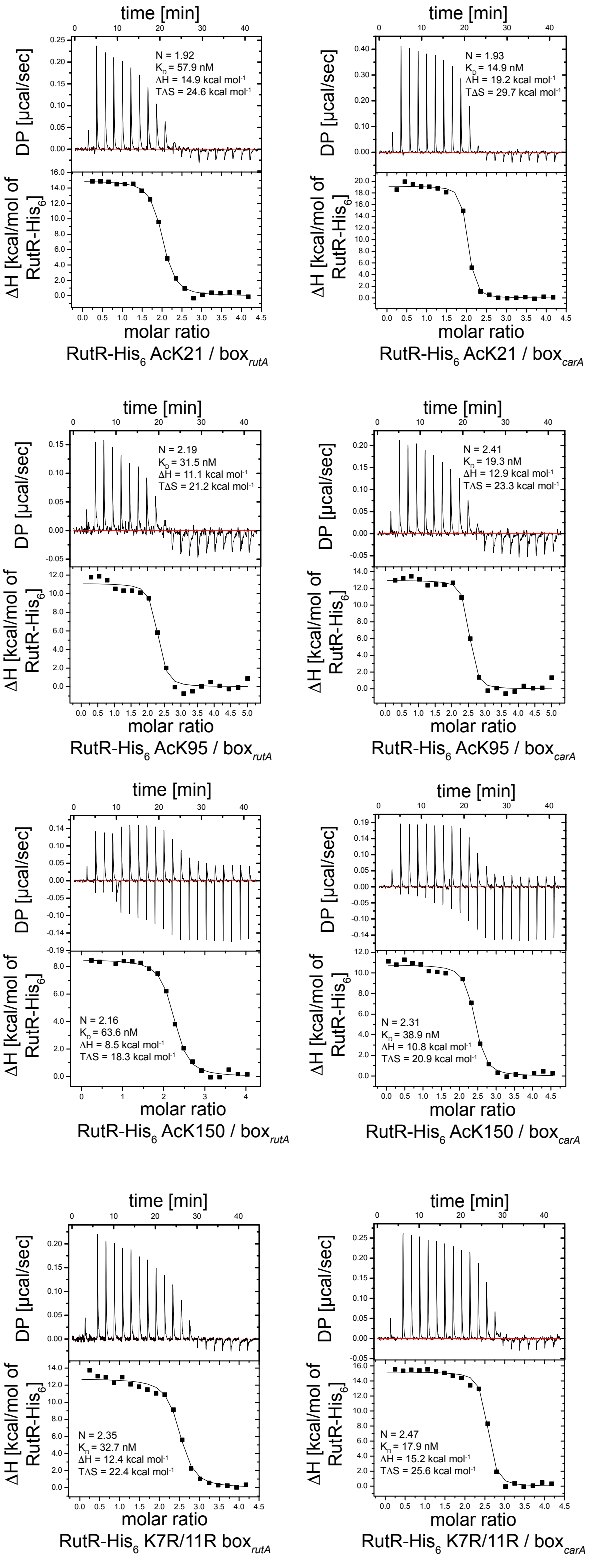


**Supplementary Figure 7: Thermodynamic characterization of the interaction of RutR AcK21, AcK95, AcK150 and K7R/K11R towards box$_{carA}$ and box$_{rutA}$ DNA.**

90-100 $\mu$M of the lysine acetylated RutR proteins were stepwise titrated from the syringe to 5 $\mu$M of box$_{carA}$ and box$_{rutA}$ DNA in the sample cell. The acetylated RutR proteins and the double mutant RutR K7R/K11R show an endothermic heat profile and a stoichiometry of 2:1, i.e. a RutR dimer binds to one box$_{carA}$ or box$_{rutA}$ dsDNA fragment. The affinities were similar to the interactions of non-acetylated RutR wild-type with box$_{carA}$ and box$_{rutA}$ DNA (Source Data file). AcK21: *n*=1; AcK95: *n*=1; AcK150: *n*=2; K7R/K11R: *n*=2. Source data are provided as a Source Data file.

a

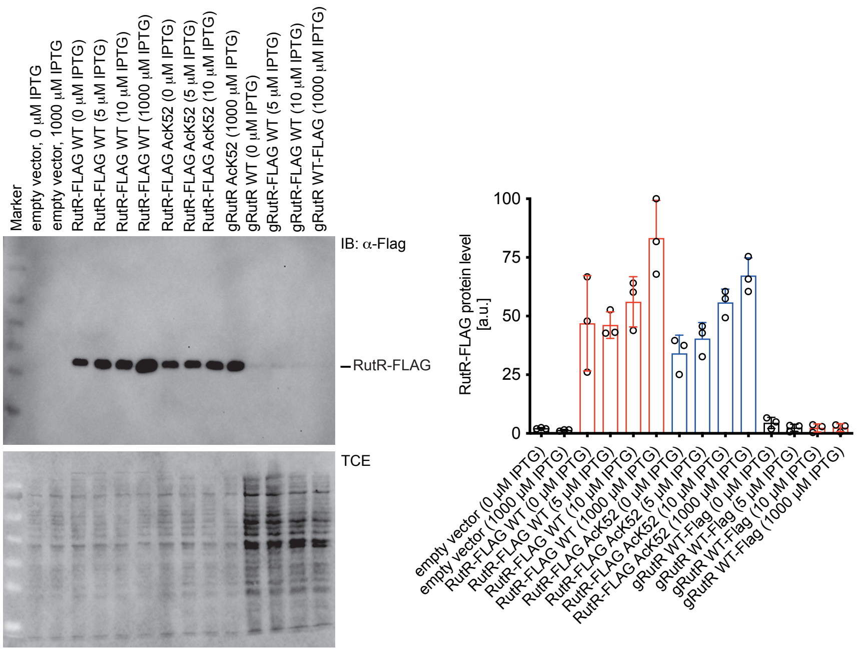


b

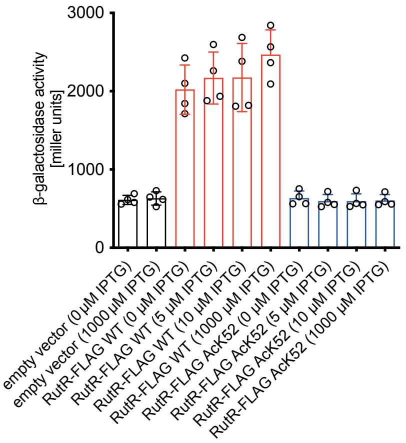


**Supplementary Figure 8: Impact of varying IPTG concentrations on the protein level of ectopically expressed *rutR* variants and RutR protein level after endogenous expression.**

**a.** For ectopic expression cells of *E. coli* U65 Δ*rutR* with a genomic $P_{carA}$-*lacZ* fusion were transformed with pRSFDuet-1/*rutR*/*ackRS3*/*MbpylT* or pRSFDuet-1/*rutRK52amber*/*ackRS3*/*MbpylT*. Expression of *rutR-FLAG* at endogenous level was achieved using *E. coli* U65 *rutR-FLAG-kanR.* As indicated, using varying IPTG concentrations (0, 5, 10 and/or 1000 $\mu$M) were applied to assess the impact of IPTG on the expression. Cells were harvested at $OD_{600}$ = 0.6. RutR-FLAG protein levels were detected by immunoblotting with an anti-FLAG-antibody. TCE staining served as loading control. For endogenous expressions (g: genomically inserted *rutR-FLAG*) double the volume of the cell lysate was loaded compared to the ectopic expressions. a.u. arbitrary units.The protein level was quantified by ImageJ software. Statistical analyses was performed using students t-test (unpaired, two-tailed). Experiments were performed in three biologically independent experiments, two of which contain two technical replicates (*n*=3). Source data including statistical analyses are provided as Source Data file.

**b.** The samples described in A for IPTG-dependent ectopic expressions of *rutR-FLAG* (encoding for non-acetylated and K52-acetylated RutR-FLAG) were analysed for β-galactosisase activity as an indicator for RutR-FLAG transcriptional regulator activity. All RutR-FLAG AcK52 samples show a statistically significant reduction in β-galactosidase activity compared to the samples of non-acetylated RutR, reaching a level similar to the empty vector controls. Notably, these effects are independent of the IPTG concentration used. Statistical analyses was performed using students t-test (unpaired, two-tailed; Source Data file). Experiments were performed in four biologically independent experiments, each with two technical replicates (*n*=4). Source data including statistical analyses are provided as Source Data file.

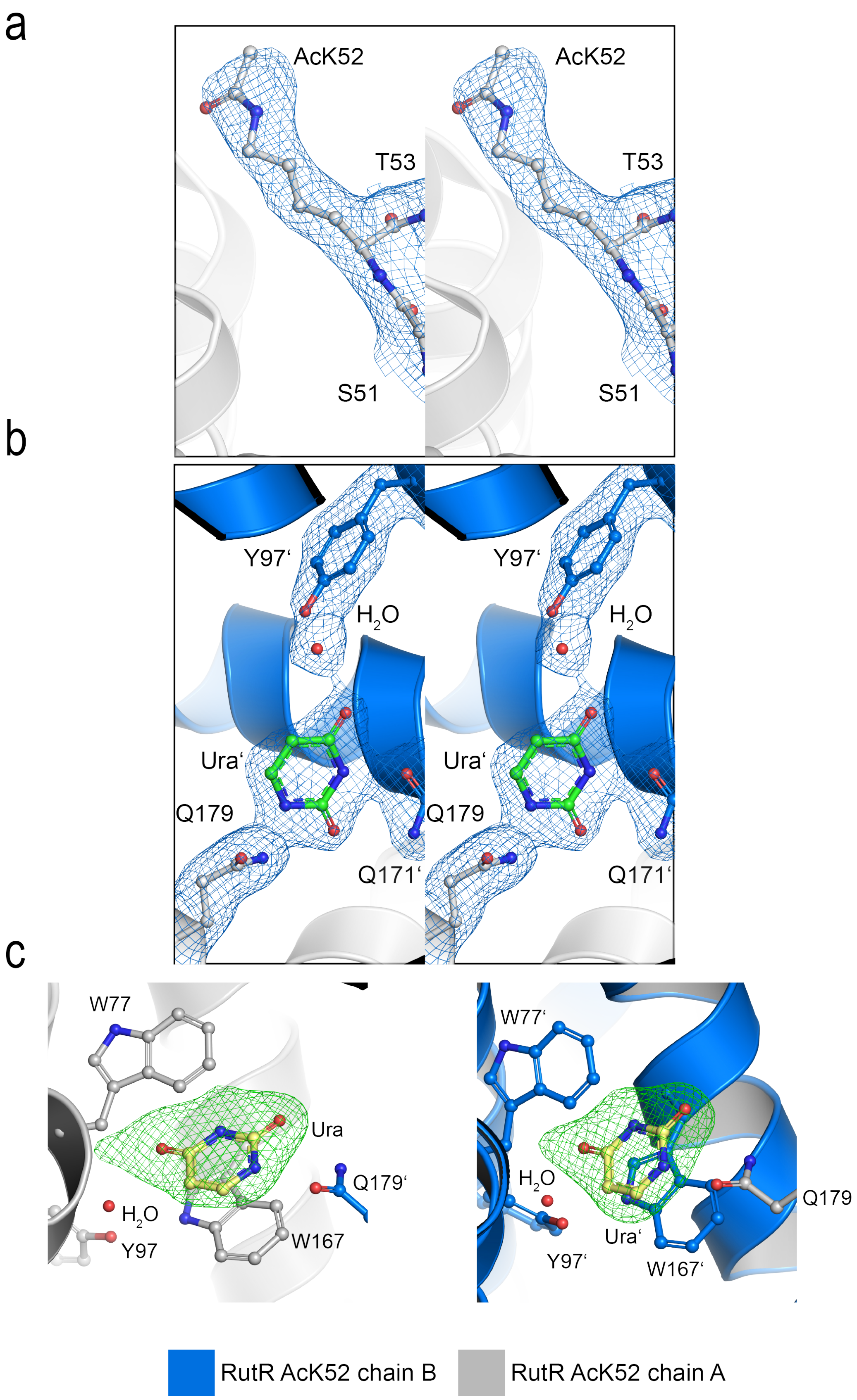


**Supplementary Figure 9: Representative electron density for the RutR AcK52•uracil structure (PDB: 6Z1B [https://doi.org/10.2210/pdb6Z1B/pdb]).**

**a,b.** Stereo figures for the RutR AcK52•uracil structure showing representative electron density of the acetyl-L-lysine AcK52 from chain A (a) and of the uracil binding site of chain B (b). Shown in blue is the electron density of the $2F_o$-$F_c$ map contoured at $1\sigma$. The stereo diagrams were prepared in wall-eyed viewing mode with PyMOL version 2.3.4[1].

**c.** $F_o$-$F_c$ omit map for uracil ligand bound in the LBD of chain A and chain B contoured at $3\sigma$. The Figure was prepared with PyMOL version 2.3.4[1].

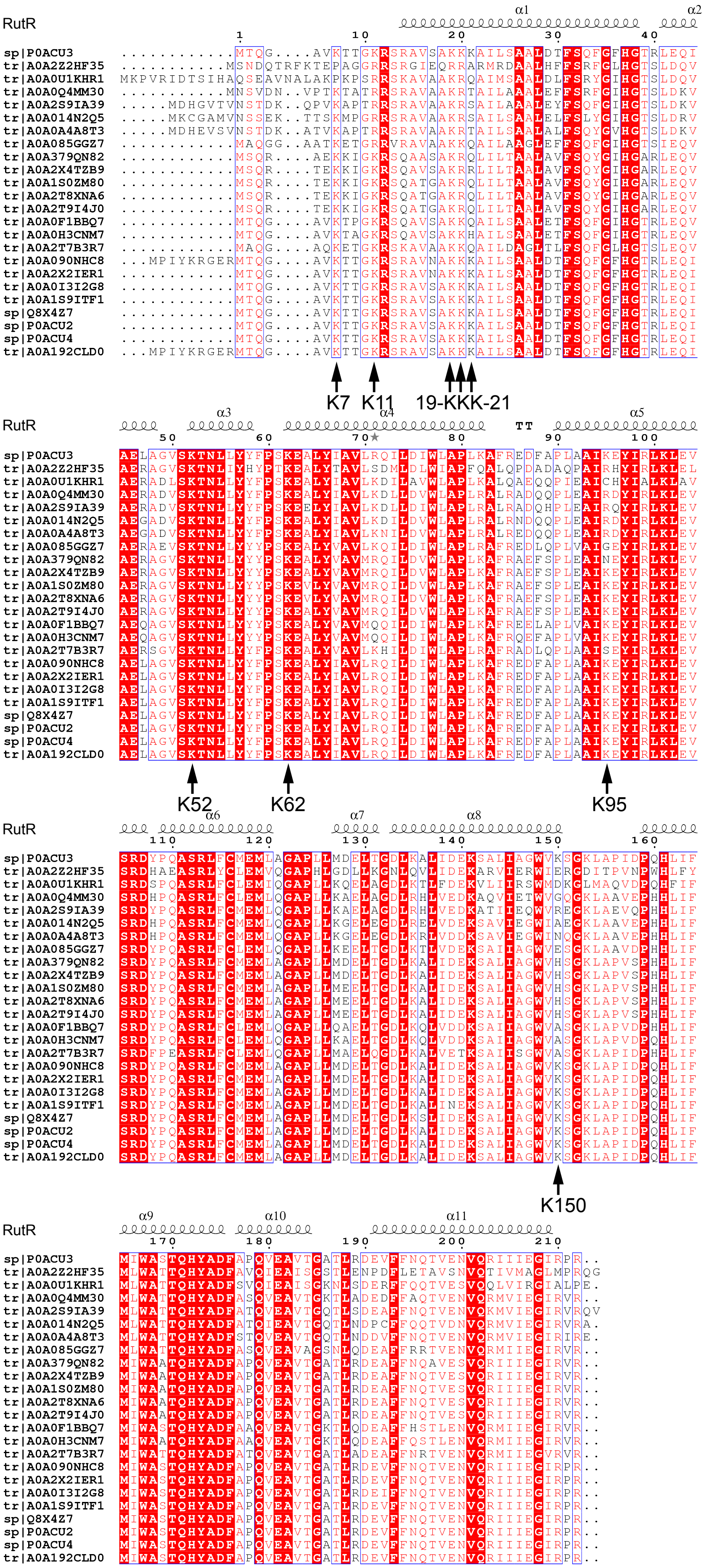
RutR
K7
K11
19-KKK-21
K52
K62
K95
K150
sp|P0ACU3
tr|A0A2Z2HF35
tr|A0A0U1KHR1
tr|A0A0Q4MM30
tr|A0A2S9IA39
tr|A0A014N2Q5
tr|A0A0A4A8T3
tr|A0A085GGZ7
tr|A0A379QN82
tr|A0A2X4TZB9
tr|A0A1S0ZM80
tr|A0A2T8XNA6
tr|A0A2T9I4J0
tr|A0A0F1BBQ7
tr|A0A0H3CNM7
tr|A0A2T7B3R7
tr|A0A090NHC8
tr|A0A2X2IER1
tr|A0A0I3I2G8
tr|A0A1S9ITF1
sp|Q8X4Z7
sp|P0ACU2
sp|P0ACU4
tr|A0A192CLD0

**Supplementary Figure 10: Primary sequence alignment of selected bacterial RutR proteins (UniProt accession numbers are shown).**

All sequences were aligned by ClustalW multiple sequence alignment (Bioedit). Most RutR proteins contain a positively-charged N-terminal tail preceding the HTH-motif. *E. coli* RutR K7, K11, K20, K52 and K62 are highly conserved. The acetylated lysines identified in this study are marked with an arrow. The secondary structure elements and numbering is shown for RutR above the sequence alignment. The alignment was created by ESPript version 3.0[2].

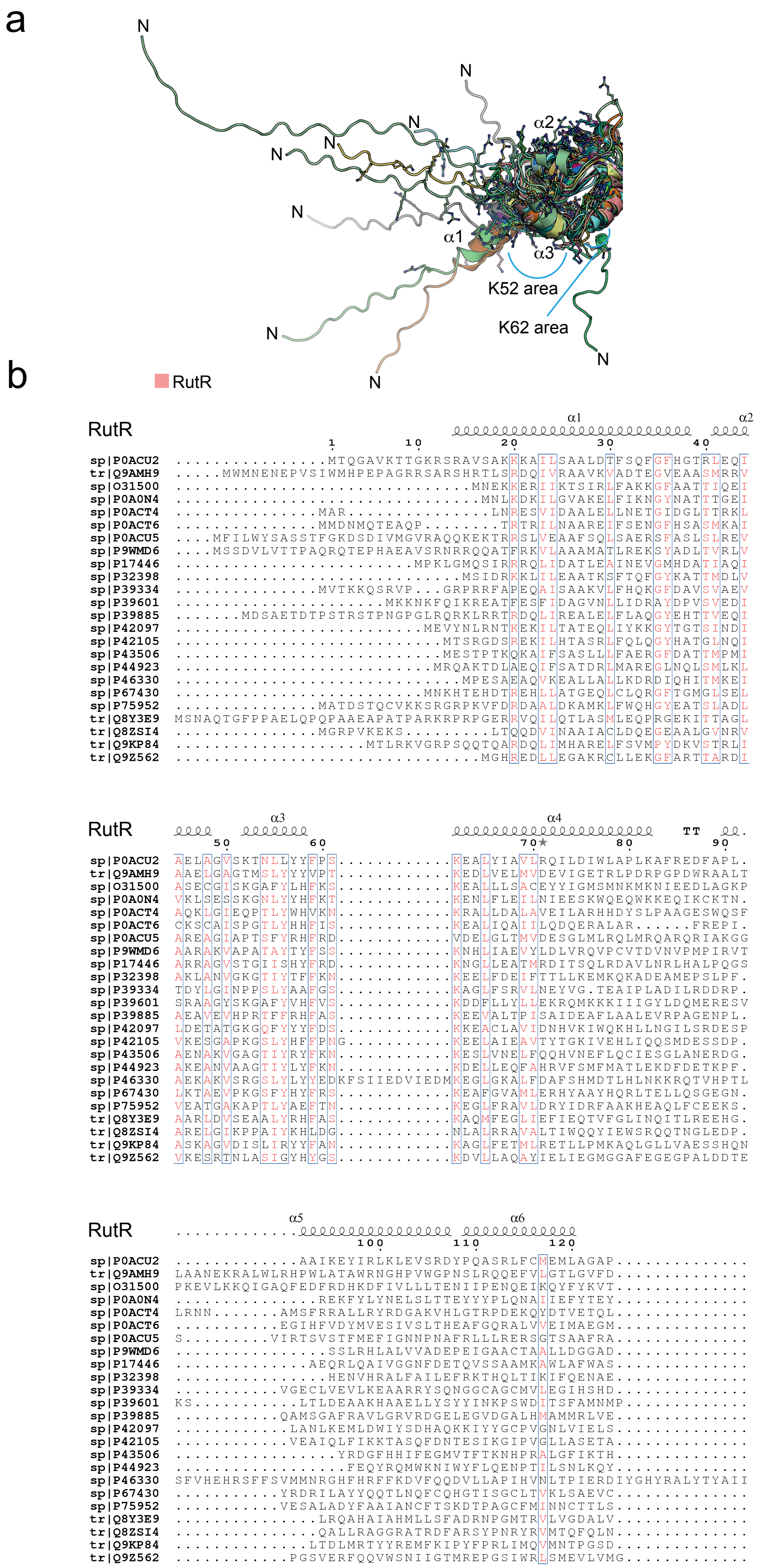
a
N
α1
α2
α3
K52 area
K62 area
b
RutR
RutR

```
RutR                 α7            α8
                     130                 140       150       160
sp|P0ACU2 .........LLMDELTGDLKALID.............EKSALIAGWVKSGKLAPIDPQHL
tr|Q9AMH9 .........LQVDELLSLIGLYNG.............YVESFVRNEVGWLEEARRTKVDM
sp|O31500 .........METDKLYRNALLVSYGEGIERYVADLSIMARGIVHSYMNVMVFNGELNIDA
sp|P0A0N4 .........YKTNSINEKMNKLEN.............KYIDAYHVIFKEGNLNGEWCIN.
sp|P0ACT4 .........RFMTENGFSLRDGLY.............AISAVSHFTLGAVLEQQEHTAAL
sp|P0ACT6 .........RNP.QVAAMLKNKHM.............TITEFVAQRMRDAQQKGEISPDI
sp|P0ACU5 .........AVAREIQHFIAELADY...........LELENHMPRAFTEAQAEAMVTIVF
sp|P9WMD6 .........PAVRAVRDRIGAEIHR............RITSAIGPGADPGTVFALEMAFF
sp|P17446 .........SMHQPMLYRLQQVSSR...........RLLSNLVSEFRRELPREQAQEAGY
sp|P32398 .........IGTMAVQEVIQKMER.............SILSYIKSKIEDGIKSGAIKPCD
sp|P39334 .........PQARDIA...VQYYH.............AAETTIYDYIARRHPQ.......
sp|P39601 ........TYKVLKKCKAYVRLYELWVEENVLYIKWLKERKLIDACIDPEYTAKIICAVL
sp|P39885 .........RTPGLLAEYLRRSEE.............MEGRLAR..IIAAREGVDLDDDF
sp|P42097 .........ALDEDFRKPLEQLFS................DLQKKIAENLSALTGLLVKQ
sp|P42105 .........LISEPLRTVCMKVFKS............WEAVFARKLMENGFAEEEAN...
sp|P43506 .........SQGTFLTEESRLAYQ.............KLVEFVCTFFREGQKQGVIRNLP
sp|P44923 .........ESLPNFKDICKNIKN.............CRWDLFCHQAQKAGLLAELS...
sp|P46330 LYWLNEDAAASPEAISQKVWELVSQKRFYWLFG.KAVPGEREKEKQIDRRVVRTREALQK
sp|P67430 .........DLSEDMRSAMDKGAR.............GVIALLSQALENGRENHCLTFCG
sp|P75952 .........PDSGDIANTLKSRHA.............MQERTLQQFLCQRQARGEIPPHC
tr|Q8Y3E9 .........GEDERLPERMAQCMD.............RIEASLKQSLRVAVTQGAWPADA
tr|Q8ZSI4 .........LQDPETSPLIQEVFH.............FLKRVLQSYNLSETKLIDAMRMV
tr|Q9KP84 .........MKKQLIEKVVLDITRP............IQETLFEKLIERGVIREGMDPQL
tr|Q9Z562 .........DRMPELRDHLGRAQR.............EAGRGIIPLLMGGREEDVTDETT
```

```
RutR           α9         α10             α11
                 170        180        190        200        210
sp|P0ACU2 IFMIWASTQHYADFAPQVEAVTGATLRDEVFFNQTVENVQRIIIEGIRPR..........
tr|Q9AMH9 REWMRRSGPYAQQLVDSGEYPMFARVLAETVAPHMG.PDQRFRSGLERLLDSIGASLDRL
sp|O31500 EEISAFIIERLDDLVQGLSRSALNPIVSKDIFNPMPAGKDQLLEDIQKVKENSTLPEDIT
sp|P0A0N4 .......DVNAVSKIAANAVNGIVTFTHEQNINERIKLMNKFSQIFLNGLSK........
sp|P0ACT4 TDRPAAPDENLPPLLREALQIMDSDDGEQAFLHGLESLIRGFEVQLTALLQIVGGDKLII
sp|P0ACT6 ......NTAMTSRLLLDLTYGVLADIEAEDLAREAS.FAQGLRAMIGGILTAS.......
sp|P0ACU5 SAGAEALDVGVEQRRQLEERLVLQLRMISKGAYYWYRREQEKTAIIPGNVKDE.......
sp|P9WMD6 G...........ALVQAGSGTFTYHEIADRLGYVVGLILAGANEPSTGGSE........
sp|P17446 G.........LAALIDGLWLRAALSGKPLDKTRANSLTRHFITQHLPTD..........
sp|P32398 P.......ELTAFVMLKLYIALIFDWEKQHPPLDKETIAGLLELYVVKGLSAN.......
sp|P39334 ......RAQCVTDFMSTVMSGLSAKAREGHSIEQLC.ATAAMAGEAIKTILEE.......
sp|P39601 DG....IIKQSYVLGQPATFRSFLDALSVFFTLDREHERPRILKSFSESEEHQ.......
sp|P39885 RPRFIVAVFKAVGRVVSREWYLRADTDLEALSVAFESALDSLRPELFADWRRPGA.....
sp|P42097 N.......LPAAHAIIAQIQGSLLLLKVTQDLNVLESNFDLLKTSFEKVGEK........
sp|P42105 .........QLGTLINSMIEGGIMLSLTNKDKTPLLLIAEQIPVLVRKKG.........
sp|P43506 ........ENALIAILFGSFMEVYEMIENDYLSLTDELLTGVEESLWAALSRQS......
sp|P44923 ........EDILFLLSLKTAINLASDAKFIDFDLKPEILESVIERSWRAIQK........
sp|P46330 AFLDVLAEKQDYAGITISDITRKSNIRRATFYDHYANKEELLQTMIQRSCAEIIDHLTIA
sp|P67430 EP......LQQAQVLYALWLGANLQAKISRSFEPLENALAHVKNIIATPAV.........
sp|P75952 ......DVTHLAEFLNCIIQGMSISAREGASLEKLM.QIAGTTLRLWPELVK........
tr|Q8Y3E9 D......IASRANLIVCVVLGRWHRYAKSGFRRSPVEGAEAQLRVLLG............
tr|Q8ZSI4 N.......AAITGFIAIEQAGLMTLERSTDASYEVMIDALVVAIQHIQKVDS........
tr|Q9KP84 CKISYLSLMIFPFIAPPALLKIHGVELSQTFLESLVEHNIQLMEQGFITRMG........
tr|Q9Z562 DTLG....TFYVTLMTGLIAQWTFDPKSAPDADALTEGLRRVIGAATGDAP.........
```

```
RutR

sp|P0ACU2 ............................................................
tr|Q9AMH9 SPPGRSAASERPALA.............................................
sp|O31500 VSLDVIEEELTQDKPRKPIIKGMLSNLAGTNDKEVERLRALILSLSQFDHKKSSL.....
sp|P0A0N4 ............................................................
sp|P0ACT4 PFC.........................................................
sp|P0ACT6 ............................................................
sp|P0ACU5 ............................................................
sp|P9WMD6 ............................................................
sp|P17446 ............................................................
sp|P32398 ............................................................
sp|P39334 ............................................................
sp|P39601 ............................................................
sp|P39885 ............................................................
sp|P42097 ............................................................
sp|P42105 ............................................................
sp|P43506 ............................................................
sp|P44923 ............................................................
sp|P46330 SSPNEFSLKEAEKALAILLSALSNMPIVHFLNREWGVPHVIPDMFKALESFYLHQQTDIH
sp|P67430 ............................................................
sp|P75952 ............................................................
tr|Q8Y3E9 ............................................................
tr|Q8ZSI4 ............................................................
tr|Q9KP84 ............................................................
tr|Q9Z562 ............................................................
```

```
RutR

sp|P0ACU2 ...................................................
tr|Q9AMH9 ...................................................
sp|O31500 ...................................................
sp|P0A0N4 ...................................................
sp|P0ACT4 ...................................................
sp|P0ACT6 ...................................................
sp|P0ACU5 ...................................................
sp|P9WMD6 ...................................................
sp|P17446 ...................................................
sp|P32398 ...................................................
sp|P39334 ...................................................
sp|P39601 ...................................................
sp|P39885 ...................................................
sp|P42097 ...................................................
sp|P42105 ...................................................
sp|P43506 ...................................................
sp|P44923 ...................................................
sp|P46330 AEKKLYAHYVSAMIIGLLLYRLDEGKAHPPEVLAREFLQFLDVKKYKVVLL
sp|P67430 ...................................................
sp|P75952 ...................................................
tr|Q8Y3E9 ...................................................
tr|Q8ZSI4 ...................................................
tr|Q9KP84 ...................................................
tr|Q9Z562 ...................................................
```

**Supplementary Figure 11: Primary sequence and structural alignment of selected TetR-related transcriptional regulators of various Gram-positive and Gram-negative bacterial strains (UniProt accession numbers are shown).**

**a.** Structural alignment of AlphaFold2 models of selected TetR-related transcriptional regulators of various bacterial strains (Supplementary Data 3) were aligned onto the HTH motif of RutR. Shown are only the N-terminal tails and the HTH-motifs. Many transcription factors carry an overrepresented number of positively-charged residues in the N-terminal tail preceding the HTH-motif and within the HTH-motif suggesting a potential to be regulated by lysine acetylation. The area, in which K52 of RutR is located (at N-terminus of $\alpha 3$) and in which K62 is located (N-terminal region of $\alpha 4$) is shown. This resembles histone-like N-terminal tails of eukaryotes and suggests a similar regulation of transcription factor DNA-binding by lysine acetylation as observed for histones in eukaryotes. Lysine and arginine side chains are shown in stick representation (Supplementary Data 3).

**b.** Sequences of all proteins listed in Supplementary Data 3 were aligned by ClustalW multiple sequence alignment (Bioedit). Several proteins contain a positively-charged N-terminal tail preceding the HTH-motif. The secondary structure elements and numbering is shown for RutR above the sequence alignment. RutR K52 and K62 are conserved in several family members. The 19-KKK-21 basic motif of RutR is also conserved in several family members. The alignment was created by ESPript version 3.0[2].

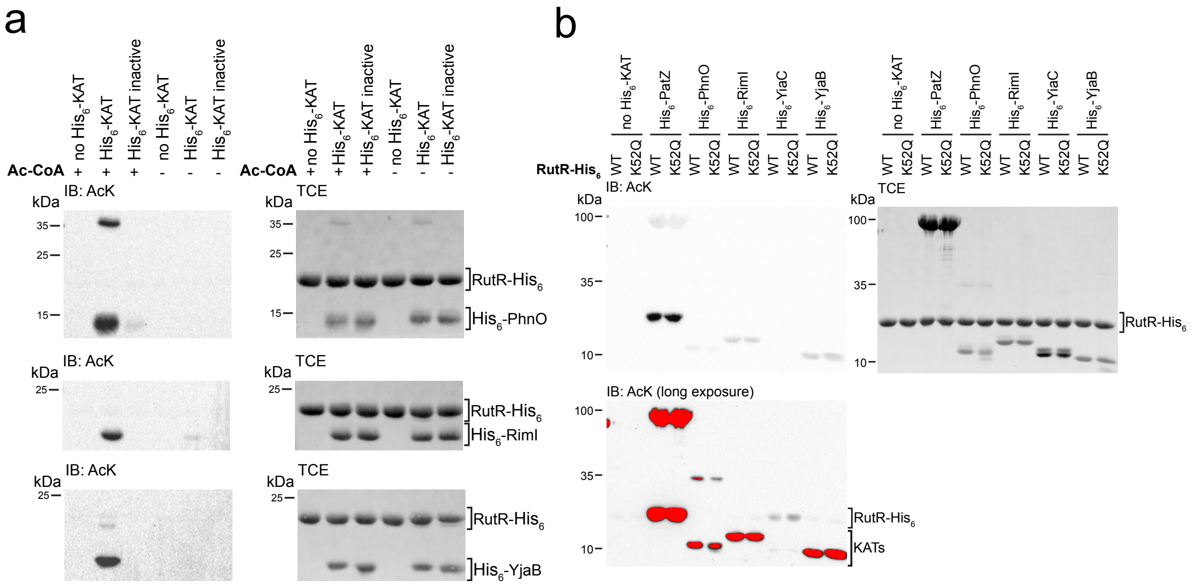


**Supplementary Figure 12: The KATs PhnO, YjaB and RimI are inactive in acetylating RutR and PatZ/YfiQ and YiaC are not able to acetylate RutR K52.**

**a.** PhnO, YjaB and RimI are not active in acetylating RutR. As described before, RutR was incubated with the respective KAT enzymes (inactive: PhnO Y128A; RimI Y115A; YjaB Y117A) in presence/absence of acetyl-coenzyme A (Ac-CoA) as indicated. The samples were subsequently analyzed in immunoblots (IB) probed with anti-AcK AB and total protein staining using 2,2,2-trichloroethanol (TCE) was used as loading control. For PhnO, YjaB and RimI an autoacetyltransferase activity is observed. One exemplary result is shown and the results were confirmed in at least three replicates ($n \geq 3$). Long exposure was conducted to visualize weak signals. Red color indicates oversaturation of signal. Source data are provided as Source Data file.

**b.** PatZ/YfiQ and YiaC are not active in acetylating RutR at K52. *In vitro* KAT activity assays with RutR and RutR K52Q show that the signal is not impaired in RutR K52Q. The samples were subsequently analyzed in immunoblots (IB) stained with anti-AcK AB. 2,2,2-trichloroethanol (TCE) staining was used as loading control. One exemplary result is shown and the results were confirmed in three replicates ($n=3$). Red indicates oversaturation of signal. Source data are provided as Source Data file.

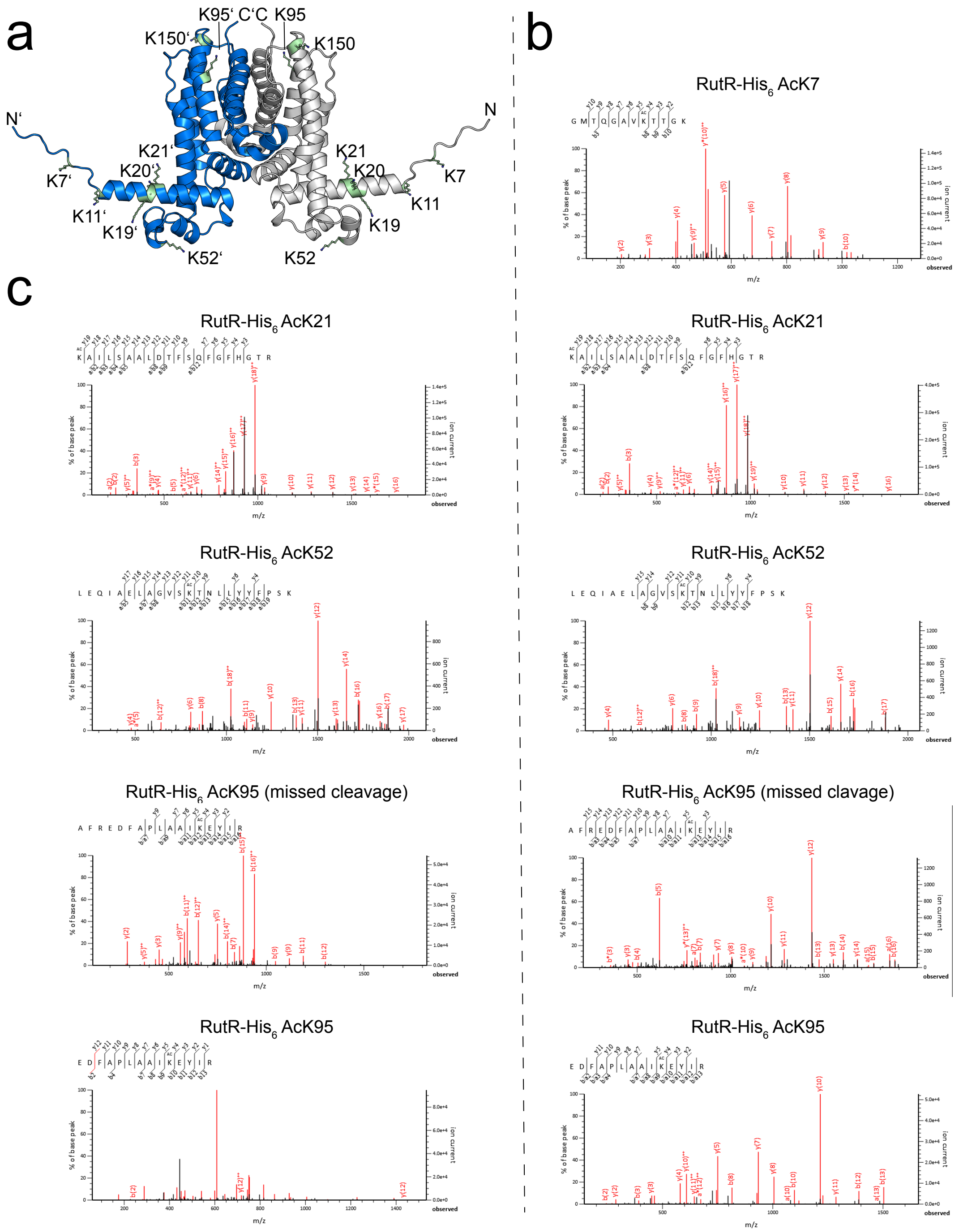


**Supplementary Figure 13: MS/MS fragmentation spectra of lysine-acetylated peptides obtained for RutR acetylated *in vitro* upon treatment with 10 mM acetyl-phosphate for 18 h.**

**a.** We show here that RutR is acetylated non-enzymatically by acetyl-phosphate at lysines in the N-terminal histone-like tail (K7, K11 and K7/K11), in the helix $\alpha$1 preceding the HTH-motif (triple K-motif, 19-KKK-21), in the HTH-motif (K52) and in the LBD (K95 and K150). The acetylation sites are shown in stick representation highlighted in green using the AlphaFold2 structure of RutR. Other reported sites reported earlier include K62 in $\alpha$4[3,4].

**b.** RutR full-length was treated for 18 h (B) with 10 mM acetyl-phosphate in 100 mM $K_2PO_4/KH_2PO_4$ pH7.5 at room temperature. Afterwards the proteins were digested with trypsin and analyzed by mass-spectrometry. The spectra obtained for acetylated peptides identified after treatment for 18 h are shown. For full-length RutR we could identify AcK7, AcK21, AcK52 and AcK95. In a study performed earlier in our group we also identified K7, K11, K19, K20, K21, K95 and K150 in full-length RutR.

**c.** RutR $\Delta$1-12 was treated for 18 h (B) with 10 mM acetyl-phosphate in 100 mM $K_2PO_4/KH_2PO_4$ pH7.5 at room temperature. Afterwards the proteins were digested with trypsin and analyzed by mass-spectrometry. The spectra obtained for acetylated peptides identified after treatment for 18 h are shown. For RutR $\Delta$1-12 we could identify AcK21, AcK52 and AcK95.

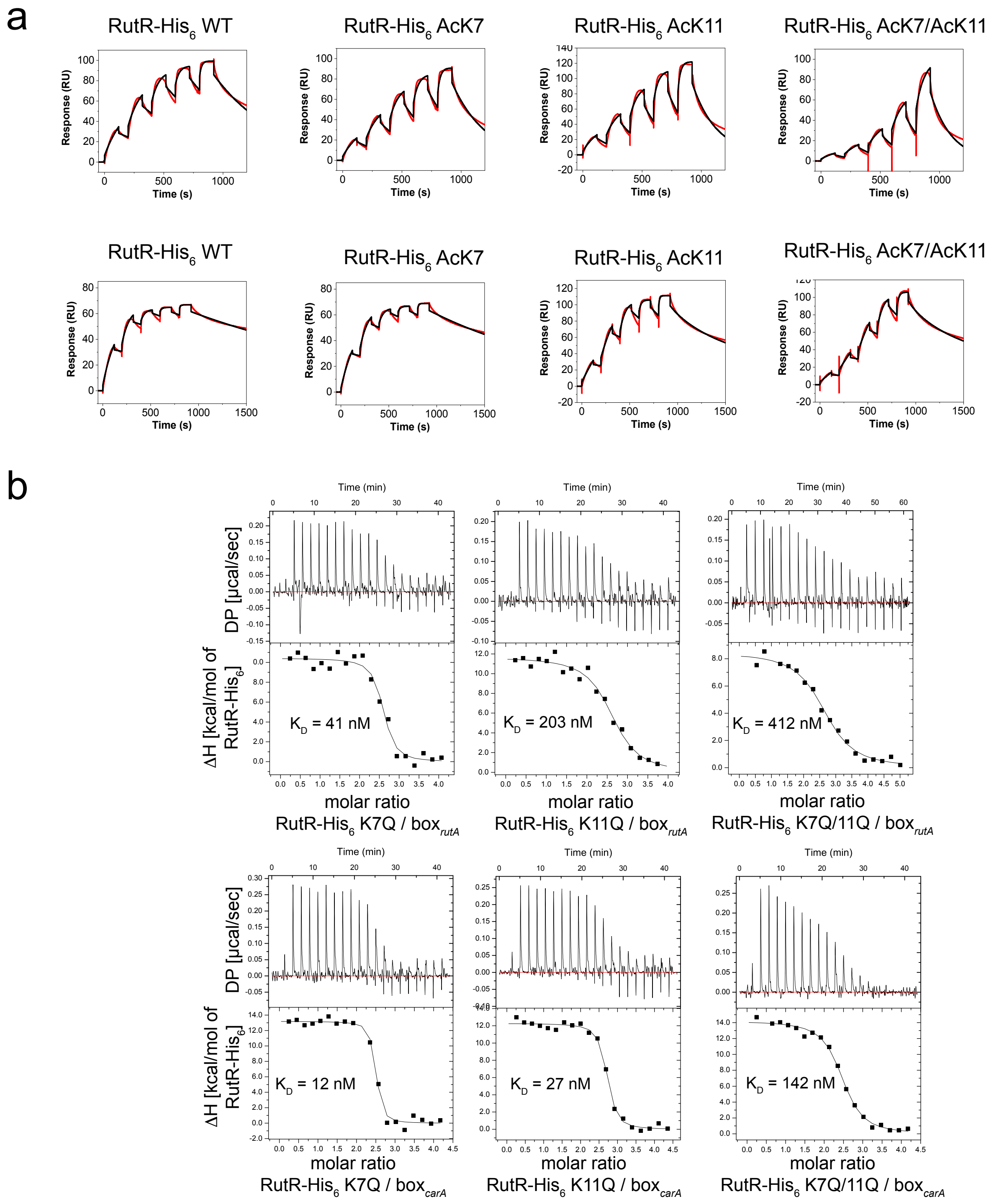


**Supplementary Figure 14: Surface plasmon resonance (SPR) data and ITC data for the interaction of acetylated and mutated RutR with dsDNA.**

**a.** Primary SPR data for the analyses of the kinetics of the interaction between non-acetylated RutR (WT) and RutR AcK7, AcK11 and AcK7/11 with $box_{rutA}$ (upper panels) as well as $box_{carA}$ (lower panels) by SPR measurements. The association rate constants ($k_{ass}$) and dissociation rate constants ($k_{diss}$) are derived. RU: response units. One representative trace is shown for each sample. Three replicates were performed (*n*=3). Source data are provided as Source Data file.

**b.** Thermodynamic characterization of the interaction of RutR K7Q, K11Q and K7Q/K11Q and $box_{carA}$ and $box_{rutA}$ by ITC. Mutation of K11Q results in decrease in the binding affinity towards both, $box_{carA}$ and $box_{rutA}$. Double mutation in RutR K7Q/K11Q results in an approximately tenfold reduction in dsDNA-binding affinity compared to RutR WT. Representative ITC diagrams are shown. Three replicates were performed (*n*=3). Source data are provided as Source Data file.

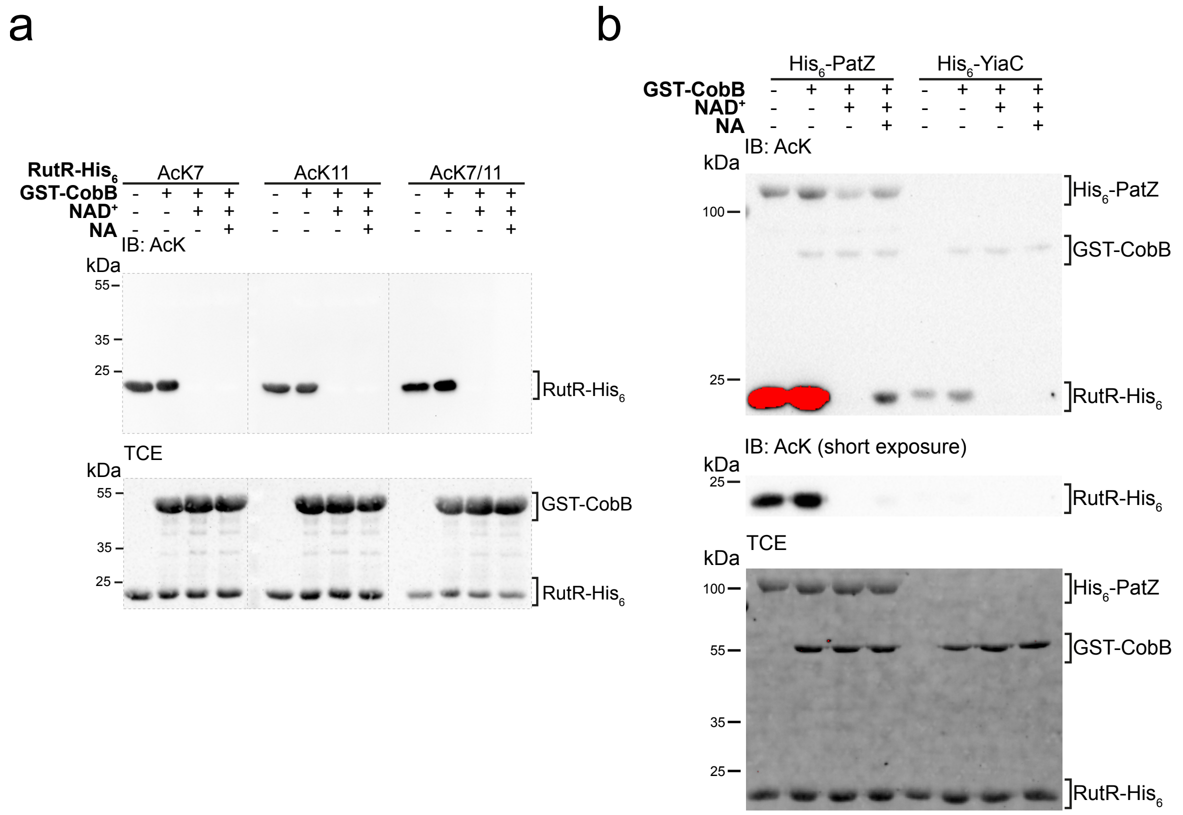


**Supplementary Figure 15: CobB-mediated deacetylation of RutR AcK7, AcK11 and AcK7/11.**

**a.** CobB-catalysed deacetylation of RutR AcK7, AcK11 and AcK7/11 is not inhibited using 10 mM nicotinamide (NA) under the reaction conditions used resulting in complete RutR deacetylation. This shows that these sites are more efficiently deacetylated by CobB compared to RutR AcK52 or AcK62. Source data are provided as Source Data file.

**b.** CobB can completely deacetylate RutR protein acetylated enzymatically by PatZ/YfiQ or YiaC. Red indicates oversaturation of the signal.

**Supplementary Table 1: *Escherichia coli* strains used in this study.**

| Strain | Genotype | Reference/construction |
|---|---|---|
| BW30270 | MG1655 *rph*$^+$ | CGSC#: 7925 |
| U65 | *rph*$^+$ *ilvG*$^+$ Δ(*araC-araBAD*) Δ(*lacI-lacZYA*) $P_{cp8}$*araE* Δ(*araH araF*)$_{FRT}$ | [5] |
| U65 rutR-FLAG-kanR | U65 *rutR-FLAG-neo-SUB11* | [6] |
| BL21 (DE3) | *B* $F^-$ *ompT gal dcm lon* $hsdS_B(r_B^- m_B^-)$ *λ(DE3 [lacI lacUV5-T7p07 ind1 sam7 nin5])* $[malB^+]_{K\text{-}12}(\lambda^S)$ | [7] |
| DH5α | $F^-$ *endA1 glnV44 thi-1 recA1 relA1 gyrA96 deoR nupG purB20 φ80dlacZΔM15 Δ(lacZYA-argF)U169, hsdR17*$(r_K^- m_K^+)$, $\lambda^-$ | [8] |

**Supplementary Table 2: Oligonucleotides used for RutR-DNA interaction studies.**

| **name** | **sequence** | **purpose** |
|---|---|---|
| rutA 50mer for | GTTTTATGTGCAACTGTTTTGACCGTTTAGTCCACTTTTTACCAGATATTTTAG | annealing to generate $box_{rutA}$ doublestranded DNA (dsDNA) |
| rutA 50mer rev | CTAAAATATCTGGTAAAAAGTGGACTAAACGGTCAAAACAGTTGCACATAAAAC | |
| carAbox46mer for | GTTTTAATGTAAATTTTGACCATTTGGTCCACTTTTTTCTGCTCGT | annealing to generate $box_{carA}$ doublestranded DNA (dsDNA) |
| carAbox46mer rev | ACGAGCAGAAAAAAGTGGACCAAATGGTCAAAATTTACATTAAAAC | |
| ctrlRutAforGDIrevSalI | CAAGAAGGAGTGGAAGGACTAAACGCGTCGACGTCGGCCATAGCGGCCGCGGAA | annealing to generate ctrl doublestranded DNA (dsDNA) |
| ctrlRutArevGDIrevSalINEU | TTCCGCGGCCGCTATGGCCGACGTCGACGCGTTTAGTCCTTCCACTCCTTCTTG | |

**Suppplementary Table 3: Thermodynamic characterization of the RutR interactions box$_{rutA}$ and box$_{carA}$ dsDNA as determined by ITC.** $K_D$ is the equilibrium dissociation constant, ΔH is the change in reaction enthalpy, ΔS the change in entropy and N the stoichiometry of the interaction. 100 μM of non-acetylated RutR/lysine-acetylated RuR/mutated RutR was titrated from the syringe into the cell containing 5 μM box$_{rutA}$ or box$_{carA}$ dsDNA, respectively. All measurements were conducted at 20°C (RutR AcK150: 25°C). Shown are the mean values of at least three measurements, except AcK21 (*n*=1), AcK95 (*n*=1), AcK150 (*n*=2), K7R/K11R (*n*=2) and the standard deviation (s.d.). Source data are provided as Source Data file.

| dsDNA | Interaction with RutR (100 μM) | $K_D$ (nM) | ΔH (kcal mol$^{-1}$) | TΔS (kcal mol$^{-1}$) | N |
|---|---|---|---|---|---|
| **box$_{rutA}$ (5 μM)** | WT | 44 ± 21 | + 10.50 ± 1.07 | + 20.41 ± 0.89 | 2.24 ± 0.10 |
| | AcK7 | 94 ± 4 | + 11.7 ± 0.69 | + 22.10 ± 1.54 | 1.80 ± 0.04 |
| | AcK11 | 95 ± 5 | + 8.75 ± 0.71 | + 18,12 ± 0.68 | 1.66 ± 0.19 |
| | AcK7/AcK11 | 251 ± 107 | + 6.46 ± 0.14 | + 15.37 ± 0.15 | 2.67 ± 0.06 |
| | AcK21 | 58 | + 14.90 | + 24.60 | 1.92 |
| | AcK95 | 32 | + 11.10 | + 21.20 | 2.19 |
| | AcK150 | 72 ± 12 | + 8.61 ± 0.14 | + 18.37 ± 0.04 | 2.14 ± 0.04 |
| | K7Q | 57 ± 20 | + 11.03 ± 0.71 | + 20.80 ± 0.56 | 2.26 ± 0.27 |
| | K11Q | 332 ± 179 | + 12.40 ± 0.70 | + 21.17 ± 0.51 | 2.37 ± 0.26 |
| | K7Q/K11Q | 540 ± 158 | + 11.18 ± 2.40 | + 19.60 ± 2.26 | 2.51 ± 0.34 |
| | K7R/K11R | 47 ± 20 | + 12.55 ± 0.21 | + 22.40 ± 0.00 | 2.39 ± 0.05 |
| | AcK52 | no binding | | | |
| | K52Q | 2424 ± 961 | + 15.83 ± 5.78 | + 23.33 ± 5.47 | 1.84 ± 0.14 |
| | K52R | 42 ± 30 | + 13.00 ± 1.47 | + 23.01 ± 1.16 | 2.01 ± 0.19 |
| | AcK62 | no binding | | | |
| | RutR Δ1-12 | 741 ± 417 | + 11.45 ± 2.03 | + 19.73 ± 1.70 | 1.96 ± 0.29 |
| **box$_{carA}$ (5 μM)** | WT | 28 ± 1 | + 15.36 ± 0.52 | + 25.48 ± 0.51 | 2.13 ± 0.07 |
| | AcK7 | 73 ± 22 | + 14.47 ± 0.47 | + 24.03 ± 0.32 | 2.43 ± 0.21 |
| | AcK11 | 82 ± 18 | + 14.17 ± 3.04 | + 23.63 ± 2.90 | 2.01 ± 0.14 |
| | AcK21 | 12 ± 5 | + 18.25 ± 1.34 | + 28.95 ± 1.06 | 2.02 ± 0.12 |
| | AcK95 | 19 | + 12.90 | + 23.30 | 2.41 |
| | AcK150 | 40 ± 7 | + 10.22 ± 0.80 | + 20.27 ± 0.84 | 2.47 ± 0.22 |
| | AcK7/AcK11 | 300 ± 131 | + 10.35 ± 4.72 | + 19.17 ± 5.05 | 3.13 ± 0.12 |
| | K7Q | 26 ± 12 | + 14.07 ± 0.76 | + 24.30 ± 0.46 | 2.41 ± 0.02 |
| | K11Q | 42 ± 16 | + 13.73 ± 1.29 | + 23.63 ± 1.10 | 2.54 ± 0.07 |
| | K7Q/K11Q | 153 ± 43 | + 15.13 ± 0.86 | + 24.30 ± 0.79 | 2.37 ± 0.10 |
| | K7R/K11R | 17 ± 1 | + 15.25 ± 0.07 | + 25.65 ± 0.79 | 2.50 ± 0.07 |
| | AcK52 | no binding | | | |
| | K52Q | 1219 ± 1130 | + 17.28 ± 7.50 | + 25.39 ± 6.96 | 1.95 ± 0.19 |
| | K52R | 61 ± 36 | + 15.35 ± 0.79 | + 25.11 ± 0.76 | 1.93 ± 0.18 |
| | AcK62 | 1124 ± 108 | +14.26 ± 1.57 | + 22.24 ± 1.57 | 1.58 ± 0.18 |
| | RutR Δ1-12 | 317 ± 50 | +15.62 ± 1.00 | + 24.34 ± 1.06 | 1.80 ± 0.14 |

**Supplementary Table 4: Data collection and refinement statistics for the RutR AcK52•uracil complex.**

| | RutR AcK52•uracil (PDB: 6Z1B [https://doi.org/10.2210/pdb6Z1B/pdb]) |
|---|---|
| ***Data collection*** | |
| Space group | $P2_12_12_1$ |
| Unit cell constants | |
| a, b, c (Å) | 48.3, 88.6, 150.0 |
| $\alpha,\beta,\gamma$ (°) | 90.0 90.0 90.0 |
| Wavelength (Å) | 1 |
| Resolution (Å)[a] | 44.27–2.25 (2.33–2.25) |
| $R_{merge}$ (%) | 9.6 (125.8) |
| $R_{meas}$ (%) | 10.5 (137,1) |
| $<I/\sigma I>$ | 10.5 (1.4) |
| Completeness (%) | 99.9 (99.8) |
| $CC_{1/2}$[b] (%) | 99.8 (51.8) |
| Multiplicity | 6.3 (6.4) |
| No. of observations | 199282 (19726) |
| No. of unique reflections | 31386 (3103) |
| ***Refinement*** | |
| Resolution (Å) | 2.25 |
| Reflections used in refinement | 31367 (3098) |
| No of test reflections | 1570 (160) |
| $R_{work}$[d] / $R_{free}$ (%)[c] | 0.1933 (0.3026)/0.2148 (0.3310) |
| **No. atoms (non-hydogen)** | 3455 |
| macromolecule | 3211 |
| ligands | 28 |
| solvent | 216 |
| **Protein residues** | 403 |
| **Ramachandran Plot (%)[d]** | |
| Most favored | 99.2 |
| Additionally allowed | 0.8 |
| Disallowed | 0.0 |
| Clashscore | 4.73 |
| **Average B-factor (Å$^2$)** | 56.6 |
| All macromolecule atoms | 56.3 |
| Ligands | 63.8 |
| Solvent molecules | 59.6 |
| **Root-mean-square deviations** | |
| Bond lengths (Å) | 0.01 |
| Bond angles (°) | 1.1 |

a: values for the highest-resolution shell in parentheses.
b: $CC_{1/2}$ correlation coefficient from[9].
c: $R_{work} = \Sigma |F_o - F_c| / \Sigma F_o$ where $F_o$ and $F_c$ are the observed and calculated structure factor amplitudes. $R_{free}$ is claculated similarly to $R_{work}$ using random 5% of working set of reflections[10].
d: MolProbity[11].

**Supplementary Table 5: Peptides and intensities detected after *in vitro* acetylation of RutR full length and N-terminally truncated RutR Δ1-12 by acetyl-phosphate (AcP).** The purified RutR proteins (20 μg) were incubated for 18 h with 10 mM acetyl-phosphate. The table shows the modified peptides detected in LC-MS/MS with absolute intensities divided by $10^6$ and modifications (K(Ac): acetylated lysine).

| intensity/$10^6$ | modifications | sequence (last residue) | Position of acetylated Lys in RutR |
|---|---|---|---|
| **RutR Δ1-12** | | | |
| 5.6 | K(Ac) | KAILSAALDTFSQFGFHGTR (40) | K21 |
| 9.7 | K(Ac) | LEQIAELAGVSKTNLLYYFPSK (62) | K52 |
| 4.5 | K(Ac) | AFREDFAPLAAIKEYIR (99) | K95 |
| 3.6 | K(Ac) | EDFAPLAAIKEYIR (99) | K95 |
| **RutR** | | | |
| 10.1 | K(Ac) | GMTQGAVKTTGK (11) | K7 |
| 18.1 | K(Ac) | KAILSAALDTFSQFGFHGTR (40) | K21 |
| 8.8 | K(Ac) | LEQIAELAGVSKTNLLYYFPSK (62) | K52 |
| 2.1 | K(Ac) | AFREDFAPLAAIKEYIR (99) | K95 |
| 25.3 | K(Ac) | EDFAPLAAIKEYIR (99) | K95 |

**Supplementary Table 6:** Additivity test of the impact of RutR acetylation using the isothermal titration calorimetry (ITC) data and the surface plasmon resonance (SPR) data. Analyzed is if the values of Gibb's free energy obtained for the interactions of $box_{rutA}$ and $box_{carA}$ dsDNA towards non-acetylated RutR (WT), RutR AcK7, RutR AcK11 by ITC and SPR studies are additive, i.e. their ΔΔG values add up to result in the ΔΔG values obtained for the interactions with double acetylated RutR AcK7/11. The values obtained correspond to the change in the Gibb's free energy, ΔG, and the difference in the change of Gibb's free energy, ΔΔG. The ΔG values were calculated from dissociation equilibrium constant, $K_D$. Source data are provided as Source Data file.

| Additivity: ITC experiments<br>RutR variant | $K_D$ | ΔG (kJ mol$^{-1}$) | ΔΔG (kJ mol$^{-1}$) | Σ ΔΔG (kJ mol$^{-1}$) |
|---|---|---|---|---|
| **RutR-box$_{rutA}$** | | | | |
| **WT** | 44*10$^{-9}$ | -41.51 | 0.00 | |
| **AcK7** | 94*10$^{-9}$ | -39.58 | 1.93 | 4.04 |
| **AcK11** | 95*10$^{-9}$ | -39.40 | 2.11 | |
| **AcK7/11** | 251*10$^{-9}$ | -37.18 | 4.33 | 4.33 |
| **RutR variant** | **$K_D$** | **ΔG (kJ mol$^{-1}$)** | **ΔΔG (kJ mol$^{-1}$)** | **Σ ΔΔG (kJ mol$^{-1}$)** |
| **RutR-box$_{carA}$** | | | | |
| **WT** | 28*10$^{-9}$ | -42,39 | 0.00 | |
| **AcK7** | 73*10$^{-9}$ | -40.14 | 2.26 | 4.84 |
| **AcK11** | 82*10$^{-9}$ | -39.81. | 2.58 | |
| **AcK7/11** | 300*10$^{-9}$ | -36.81 | 5.58 | 5.58 |

| Additivity: SPR experiments<br>RutR variant | $K_D$ | ΔG (kJ mol$^{-1}$) | ΔΔG (kJ mol$^{-1}$) | Σ ΔΔG (kJ mol$^{-1}$) |
|---|---|---|---|---|
| **RutR-box$_{rutA}$** | | | | |
| **WT** | 4.27*10$^{-9}$ | -47.77 | 0.00 | |
| **AcK7** | 9.06*10$^{-9}$ | -45.91 | 1.86 | 4.91 |
| **AcK11** | 1.46*10$^{-8}$ | -44.72 | 3.05 | |
| **AcK7/11** | 7.62*10$^{-8}$ | -40.63 | 7.14 | 7.14 |
| **RutR variant** | **$K_D$** | **ΔG (kJ mol$^{-1}$)** | **ΔΔG (kJ mol$^{-1}$)** | **Σ ΔΔG (kJ mol$^{-1}$)** |
| **RutR-box$_{carA}$** | | | | |
| **WT** | 4.69*10$^{-10}$ | -53.52 | 0.00 | |
| **AcK7** | 7.60*10$^{-10}$ | -52.05 | 1.20 | 3.55 |
| **AcK11** | 1.21*10$^{-9}$ | -50.90 | 2.35 | |
| **AcK7/11** | 3.91*10$^{-9}$ | -47.99 | 5.26 | 5.26 |

**Supplementary Table 7:** Single values for the kinetic characterization of the interaction of $box_{rutA}$ and $box_{carA}$ dsDNA towards non-acetylated RutR (WT), RutR AcK7, RutR AcK11 and double acetylated RutR AcK7/11 by surface plasmon resonance studies. The values obtained correspond to the association rate constant, $k_{ass}$, the dissociation rate constant, $k_{diss}$, and the calculated dissociation equilibrium constant, $K_D$. $R_{max}$ shows the maximum of obtained resonance units (RU). For $k_{ass}$, $k_{diss}$ and $K_D$, the mean values are calculated. Experiments were performed in three biologically independent experiments (*n*=3).

| RutR | $k_{ass}$ ($M^{-1}s^{-1}$) | mean $k_{ass}$ ($M^{-1}s^{-1}$) | $k_{diss}$ ($s^{-1}$) | mean $k_{diss}$ ($s^{-1}$) | $K_D$ (M) | mean $K_D$ (M) | $R_{max}$ (RU) |
|---|---|---|---|---|---|---|---|
| **RutR-$box_{rutA}$** | | | | | | | |
| **WT** | 5.86*10^5 | 4.95 ± 1.00*10^5 | 1.87*10^-3 | 2.04 ± 0.15*10^-3 | 3.19*10^-9 | 4.27 ± 1.19*10^-9 | 88.58 |
| | 5.11*10^5 | | 2.09*10^-3 | | 4.09*10^-9 | | 84.46 |
| | 3.88*10^5 | | 2.15*10^-3 | | 5.54*10^-9 | | 85.81 |
| **AcK7** | 4.43*10^5 | 3.88 ± 0.73*10^5 | 3.37*10^-3 | 3.42 ± 0.05*10^-3 | 7.61*10^-9 | 9.06 ± 1.97*10^-9 | 84.14 |
| | 4.16*10^5 | | 3.44*10^-3 | | 8.26*10^-9 | | 82.84 |
| | 3.06*10^5 | | 3.46*10^-3 | | 1.13*10^-8 | | 85.77 |
| **AcK11** | 4.16*10^5 | 3.71 ± 0.56*10^5 | 4.93*10^-3 | 5.29 ± 0.32*10^-3 | 1.18*10^-8 | 1.46 ± 0.29*10^-8 | 105.5 |
| | 3.88*10^5 | | 5.53*10^-3 | | 1.43*10^-8 | | 106.2 |
| | 3.09*10^5 | | 5.42*10^-3 | | 1.76*10^-8 | | 105.4 |
| **AcK7/11** | 1.13*10^5 | 8.42 ± 3.16*10^4 | 6.24*10^-3 | 5.80 ± 0.38*10^-3 | 5.51*10^-8 | 7.62 ± 3.03*10^-8 | 120.1 |
| | 8.92*10^4 | | 5.59*10^-3 | | 6.27*10^-8 | | 124.5 |
| | 5.04*10^4 | | 5.57*10^-3 | | 1.11*10^-7 | | 161.4 |
| **RutR-$box_{carA}$** | | | | | | | |
| **WT** | 1.09*10^6 | 9.63 ± 1.48*10^5 | 3.66*10^-4 | 4.38 ± 0.65*10^-4 | 3.35*10^-10 | 4.69 ± 1.40*10^-10 | 70.04 |
| | 9.97*10^5 | | 4.57*10^-4 | | 4.58*10^-10 | | 61.78 |
| | 8.01*10^5 | | 4.92*10^-4 | | 6.15*10^-10 | | 63.22 |
| **AcK7** | 9.10*10^5 | 8.64 ± 0.52*10^5 | 6.04*10^-4 | 6.53 ± 0.54*10^-4 | 6.64*10^-10 | 7.60 ± 1.09*10^-10 | 63.81 |
| | 8.75*10^5 | | 6.45*10^-4 | | 7.37*10^-10 | | 60.07 |
| | 8.08*10^5 | | 7.10*10^-4 | | 8.79*10^-10 | | 62.25 |
| **AcK11** | 1.13*10^6 | 1.24 ± 0.13*10^6 | 1.60*10^-3 | 1.48 ± 0.13*10^-3 | 1.42*10^-9 | 1.21 ± 0.23*10^-9 | 100.3 |
| | 1.38*10^6 | | 1.34*10^-3 | | 9.69*10^-10 | | 107.5 |
| | 1.20*10^6 | | 1.50*10^-3 | | 1.25*10^-9 | | 100.8 |
| **AcK7/11** | 6.22*10^5 | 5.69 ± 0.67*10^5 | 2.09*10^-3 | 2.19 ± 0.11*10^-3 | 3.36*10^-9 | 3.91 ± 0.68*10^-9 | 99.32 |
| | 5.92*10^5 | | 2.18*10^-3 | | 3.69*10^-9 | | 96.01 |
| | 4.94*10^5 | | 2.31*10^-3 | | 4.67*10^-9 | | 94.62 |

**Supplementary Table 8: Oligonucleotides used for cloning of protein coding sequences.**

| name | sequence | purpose |
|---|---|---|
| Gi_RutRdel2-12_His_RSF_fo | ACTTTAATAAGGAGATATACCATGGGCTCGCGCGCAGTAAGCG | generation of *rutR Δ1-12* fragment |
| Gi_Xo_RSF_RutR_r | CAGCGGTTTCTTTACCAGACTCGAGTTAACGTGGTCGAATCCCC | |
| QC RutRK52RFwd | GCGGGTGTTTCAAGAACCAATCTGCTGTATTAC | mutagenesis *rutR K52R* |
| QC RutRK52RRev | GTAATACAGATTGGTTCTTGAAACACC CGCCAAC | |
| QC RutRK52QFwd | GCGGGTGTTTCAGAAACCAATCTGCTGTATTAC | mutagenesis *rutR K52Q* |
| RutR K52Q rev | GATTGGTTTGTGAAACACCCGCCAACTCTG | |
| QC_RutR_AcK21_fo | GAAGAAATAGGCGATTCTTAGCGCAGCACTGGAC | mutagenesis *rutR K21amb* |
| QC_RutR_AcK21_rev | GTCCAGTGCTGCGCTAAGAATCGCCTATTTCTTC | |
| QC_RutR_AcK95_fo | GCGATCTAGGAGTACATCCGTCTGAAGCTG | mutagenesis *rutR K95amb* |
| QC_RutR_AcK95_rev | GTACTCCTAGATCGCCGCCAGCGGGGCGAAATC | |
| QC_RutR_AcK150_fo | GTTGGGTCTAGAGCGGCAAACTCGCGCCGATTG | mutagenesis *rutR K150amb* |
| QC_RutR_AcK150_rev | GCCGCTCTAGACCCAACCGGCAATCAGCGCCG | |
| PatflBamfor | GGTGGTGGATCCGATGAGTCAGCGAGGACTGGAAGC | genomic cloning *patZ/yfiQ* |
| PatflXhoRev | GGTGGTCTCGAGTCATGATTCCTCGCGCTGGGC | |
| PhnOflRSFBam for | GGTGGTGGATCCGATGCCTGCTTGTGAGCTTC | genomic cloning *phnO* |
| PhnOflRSFXho rev | GGTGGTCTCGAGTTACAGCGCCTTGGTGAAGC | |
| RimIflRSFBam for | GGTGGTGGATCCGATGAACACGATTTCTTCCCTC | genomic cloning *rimI* |
| RimIflRSFXho rev | GGTGGTCTCGAGTTACATACTGATTGGCAACGC | |
| YiaCflRSFBam for | GGTGGTGGATCCGATGATTCGGGAAGCGCAAC | genomic cloning *yiaC* |
| YiaCflRSFXho rev | GGTGGTCTCGAGTTACAGCGTTTGAACCACCG | |
| YjaBflRSFBam for | GGTGGTGGATCCGATGGTTATTAGTATTCGCCGCTCAC | genomic cloning *yjaB* |
| YjaBflRSFXho rev | GGTGGTCTCGAGTTACGCCCCCACATACG | |
| PatZ E809A for 3 | CATCGATGCCGCATTTGCTGTACTGGTTCGCTCGGATCTCAAAG | mutagenesis *patZ/yfiQ E809A* |
| PatZ E809A rev 3 | GTACAGCAAATGCGGCATCGATGTTATCAGGATCGGAAATCG | |
| QC_PhnO_Y128A_fo | CGTTTCGCTCTGCGCGAAGGCTACGAG | mutagenesis *phnO Y128A* |
| QC_PhnO_Y128A_rev | CGCAGAGCGAAACGGTGCGCGTCGTG | |
| QC_RimI_Y115A_fo | CCCTGGCCGAAAGTTTAGGCTTTAACGAG | mutagenesis *rimI Y115A* |
| QC_RimI_Y115A_rev | CTTTCGGCCAGGGCAATGGCGGCAGCGTTTG | |
| QC_YiaC_Y115A_fo | GATAAATTTTGCCCAGGCACAGGGTTTTCACATTGTC | mutagenesis *yiaC Y115A* |
| QC_YiaC_Y115A_rev | TGTGCCTGGGCAAAATTTATCGCCGGTTGATTTTTTTG | |
| QC_YjaB_Y117A_fo | GGGTTCGCTAAGAAGGTGGGTTTTAAGGTTACGGGAC | mutagenesis *yjaB Y117A* |
| QC_YjaB_Y117A_rev | CACCTTCTTAGCGAACCCAACCGCCTGCTCATTTTG | |
| pET-RP | CTAGTTATTGCTCAGCGG | sequencing of pRSFDuet-1 constructs |
| T7 | TAATACGACTCACTATAGGG | |
| Seq Pat T279 for | CCCTTAGCCATATGCGC | sequencing of *patZ/yfiQ* constructs |

**Supplementary Table 9: Oligonucleotides used for bacterial strain construction.**

| name | sequence | purpose |
|---|---|---|
| DelrutRDatsenkofor | CGGGCGGGGGTTTTGCTATCCTGTTGCC AATCTACAAGAGGGGAGAGCGCATGGTG TAGGCTGGAGCTGCTTCG | generation of fragment for genomic deletion of *rutR* from pKD3 |
| DelrutRDatsenkorev | CTATAGGTTAAACAGGTAGCCGGAGGAT GTTACAACCTCCTCCGGCATCTTTACATA TGAATATCCTCCTTAGTTCCTATTCC | |
| DWRutRFLAGforCTer m | GAGGTATTTTTCAATCAAACGGTTGAAAA CGTGCAGCGGATTATTATTGAGGGGATTC GACCACGTGGATCATCAGGAGACTACAA AGACCATGACGGTG | generation of fragment for genomic insertion of FLAG tag C-terminal of *rutR* from pSUB11 |
| DWRutRFLAGrevCTe rm | CCAGCTTAATGACTATAGGTTAAACAGGT AGCCGGAGGATGTTACAACCTCCTCCGG CATCTTTACATATGAATATCCTCCTTAGTT CCTATTCC | |
| RutR_fo_Test | CTGCATCCTGCATATCCTTTTCAG | control of genomic *rutR* manipulations |
| RutR_rev_Test | ACCGTTTGATGCGGTGATCTTCCAC | |
| CobB_fo_Test | CATTGAACGCGCGCGCCACGGTGATG | control of genomic *cobB* deletion |
| CobB_rev_Test | ACCAGAATATCGCCACTCTGGGCAG | |
| PromcarABforSalI | ACGCACGCGTCGACGTTGAGTGGTAAGG AAAGCGG | cloning of Prom$_{carA}$ into pKESL253 |
| ArevXbaI | CTAGCTAGTCTAGAATCCTGCATATCCTT TTCAGCCGCG | |
| S93 | CCGGGCCGACAACAAAGTCA | analysis of *attB* integration |
| T334 | TGGCGAAGTAATCGCAACATCC | |
| S95 | CATATGGGGATTGGTGGCGA | |
| T912 | GCTGGTGGCACTGGGTAGTTGTTA | |
| S118 | TGCGGGCCTCTTCGCTATTA | |
| S164 | GAGCAGGGGAATTGATCCGGTGGA | |
| M13-FP | TGTAAAACGACGGCCAGT | sequencing of pKESL253 |
| pIRES-RP | TATAGACAAACGCACACCG | |

**Supplementary Table 10: Plasmids used for bacterial strain construction.**

| name | purpose | origin |
|---|---|---|
| pKD3 | generation of fragment for genomic deletion of *rutR* with oligonucleotides DelrutRDatsenkofor and DelrutRDatsenkorev | [12] |
| pKD46 | expression of lambda red genes for recombineering | [12] |
| pCP20 | expression of Flp recombinase for deletion of resistance genes | [13] |
| pSUB11 | generation of fragment for genomic insertion of FLAG tag C-terminal of *rutR* with oligonucleotides DWRutRFLAGforCTerm and DWRutRFLAGrevCTerm | [14] |
| pLDR8 | expression of integrase for *attB* integration | [15] |
| pKESL253 | template for generation of transcriptional $P_{carA}$-*lacZ* fusion with oligonucleotides PromcarABforSalI and PromRutArevXbaI | derived of pKES268 [16], includes 19 bp instead of 14 bp encompassing native ribosome binding site of *lacZ* and lacks a HindIII site in the MCS – |
| pKESL253-$P_{carA}$ | generation of ori-less circles of for *attB* integration of $P_{carA}$-*lacZ* fusion | cloning from pKESL253 with oligonucleotides PromcarABforSalI and PromRutArevXbaI |

**Supplementary Table11: PCRs for analyses of chromosomal integrations into *attB* site.**

| oligonucleotide pair | expected fragment size [bp] | purpose |
|---|---|---|
| S93 – S164 | 563 | control *attB/P'* site |
| S95 – T912 | 718 | control *attP/B'* site |
| S95 – S164 | 581 | exclusion of dimer integration |
| T334 – S118 | 596 | $P_{carA}$ specific size |

**Supplementary Table 12: Oligonucleotides used for gene expression analysis by quantitative PCR.**

| name | sequence | purpose |
|---|---|---|
| qPCR16SrRNAfor | GGTGTAGCGGTGAAATGCGTAGAG | detection of *16SrRNA* gene expression |
| qPCR16SrRNArev | CTCAAGGGCACAACCTCCAAGTC | |
| qPCRrpoDfor | GACGAAGAAGATGGCGATGACGAC | detection of *rpoD* gene expression |
| qPCRrpoDrev | TTCCTGAGCGGTAGCGTGACTG | |
| qPCRrutRfor2 | GATTTGGGCTTCCACTCAACATTAC | detection of *rutR* gene expression |
| qPCRrutRrev2 | CTGCACGTTTTCAACCGTTTG | |
| qPCRrutAfor | CGACACTGGCGTAAGAACCGAC | detection of *rutA* gene expression |
| qPCRrutArev | GCGGATGAAGAGGCGTTAAGC | |
| qPCRcarAfor | GCATCTTCCTCTCCAACGGTCC | detection of *carA* gene expression |
| qPCRcarArev | GACCGAGACAGATGCCGAATACC | |
| qPCRlacZfor | CAGGGTGAAACGCAGGTCGC | detection of *lacZ* gene expression |
| qPCRlacZrev | CTTCAATCAGCGTGCCGTCG | |
| qPCRglxRfor | TGCTCGCCAGGTAACGGAAG | detection of *glxR* gene expression |
| qPCRglxRrev | CATATCAACAATGGTTTTGCCCTTCAG | |
| qPCRgadCfor | GCTCACATTACTTGGATTCTTTGCCATAAC | detection of *gadC* gene expression |
| qPCRgadCrev | CATTTCCGCAGCACAAAGTCC | |

## Supplementary References


1. The PyMOL Molecular Graphics System, Version 2.3, Schrödinger, LLC. (2019).
2. Robert, X. & Gouet, P. Deciphering key features in protein structures with the new ENDscript server. *Nucleic Acids Res* **42**, W320-4 (2014).
3. Kremer, M., Kuhlmann, N., Lechner, M., Baldus, L. & Lammers, M. Comment on 'YcgC represents a new protein deacetylase family in prokaryotes'. *Elife* **7**(2018).
4. Tu, S. et al. YcgC represents a new protein deacetylase family in prokaryotes. *Elife* **4**(2015).
5. Breddermann, H. & Schnetz, K. Correlation of Antagonistic Regulation of leuO Transcription with the Cellular Levels of BglJ-RcsB and LeuO in Escherichia coli. *Front Cell Infect Microbiol* **6**, 106 (2016).
6. Miller, J.H. A short course in bacterial genetics: A laboratory manual for Escherichia coli and related bacteria. *Cold Spring Harbor, NY, USA: Cold Spring Harbor Laboratory Press* (1992).
7. Studier, F.W. & Moffatt, B.A. Use of bacteriophage T7 RNA polymerase to direct selective high-level expression of cloned genes. *J Mol Biol* **189**, 113-30 (1986).
8. Hanahan, D. DNA Cloning: A Practical Approach. *Glover, D. M. (ed.)* **1**, 109 (1985).
9. Diederichs, K. & Karplus, P.A. Better models by discarding data? *Acta Crystallogr D Biol Crystallogr* **69**, 1215-22 (2013).
10. Brunger, A.T. Free R value: cross-validation in crystallography. *Methods Enzymol* **277**, 366-96 (1997).
11. Chen, V.B. et al. MolProbity: all-atom structure validation for macromolecular crystallography. *Acta Crystallogr D Biol Crystallogr* **66**, 12-21 (2010).
12. Datsenko, K.A. & Wanner, B.L. One-step inactivation of chromosomal genes in Escherichia coli K-12 using PCR products. *Proc Natl Acad Sci U S A* **97**, 6640-5 (2000).
13. Cherepanov, P.P. & Wackernagel, W. Gene disruption in Escherichia coli: TcR and KmR cassettes with the option of Flp-catalyzed excision of the antibiotic-resistance determinant. *Gene* **158**, 9-14 (1995).
14. Uzzau, S., Figueroa-Bossi, N., Rubino, S. & Bossi, L. Epitope tagging of chromosomal genes in Salmonella. *Proc Natl Acad Sci U S A* **98**, 15264-9 (2001).
15. Diederich, L., Rasmussen, L.J. & Messer, W. New cloning vectors for integration in the lambda attachment site attB of the Escherichia coli chromosome. *Plasmid* **28**, 14-24 (1992).
16. Salscheider, S.L., Jahn, A. & Schnetz, K. Transcriptional regulation by BglJ-RcsB, a pleiotropic heteromeric activator in Escherichia coli. *Nucleic Acids Res* **42**, 2999-3008 (2014).
