# Supplementary material for: Bacteria employ lysine acetylation of transcriptional regulators to adapt gene expression to cellular metabolism
Source: Nat Commun. 2024 Feb 23;15:1674. doi: 10.1038/s41467-024-46039-8 (PMC10891134; doi:10.1038/s41467-024-46039-8)
Supplement: Supplementary file 3 — Description of Additional Supplementary Files [file 41467_2024_46039_MOESM3_ESM.pdf]

Description of Additional Supplementary Files:

Supplementary Data 1: Structural alignments of related transcriptional regulators of the TetR-family.

Structural alignments were performed using the AlpphaFold2 models of the TetR-family transcriptional regulators shown below. The alignment was done by superimposing the helix-turn-helix motifs onto the HTH-motif of RutR. The r.m.s.d. values shown the close structural similarities. For the HTH-motif of Q8Y3E9, the alignment was done on the whole RutR structure as shown. The residues at the important positions of RutR K52 and K62 were listed. Moreover, residues in the N-terminal tail preceding the 19-KKK-21 motif in RutR up to the analogous position corresponding to K62 in RutR were shown. This shows that the N-terminal tails of the TetR-related transcriptional regulators carry multiple positively-charged residues, i.e. R and K. Diverse TetR-related transcription factors from different Gram-positive and Gram-negative bacterial species were analyzed.

Supplementary Data 2: Peptides and intensities detected after *in vitro* acetylation of RutR (10 µM) by KATs by LC-MS/MS.

The table shows the peptides initially detected in LC-MS/MS with absolute intensities divided by 10^6 and modifications (M(Ox): oxidized methionine; N-t.(Ac): acetylated N-terminus; K(Ac): acetylated lysine). Means ± standard deviations from three independent biological replicates are shown (*n*=3). The table corresponds to Figure 7b.

Supplementary Data 3: Peptides and intensities detected after *in vitro* acetylation of RutR (10 µM) by acetyl-phosphate (AcP).

The table shows the peptides initially detected in LC-MS/MS with absolute intensities divided by 10^6 and modifications (M(Ox): oxidized methionine; N-t.(Ac): acetylated N-terminus; K(Ac): acetylated lysine). Means ± standard deviations from three independent biological replicates are shown (*n*=3).

Supplementary Data 4: Plasmids used for protein expression.

Supplementary Data 5: Chemical structures for L-lysine, N-(ε)-acetyl-L-lysine (AcK) in a protein context, of a protein's α-amino group and of an acetylated N-(α)-acetylated amino group.
